# A tumor-associated heparan sulfate-related glycosaminoglycan promotes the generation of functional regulatory T cells

**DOI:** 10.1038/s41423-023-01096-9

**Published:** 2023-11-22

**Authors:** Leticia Martín-Cruz, Marcos Viñuela, Ioanna Kalograiaki, Alba Angelina, Paola Oquist-Phillips, Irene Real-Arévalo, Francisco Javier Cañada, José Ignacio Tudela, Luis Moltó, Jesús Moreno-Sierra, José Luis Subiza, Oscar Palomares

**Affiliations:** 1 Department of Biochemistry and Molecular Biology, School of Chemistry, Complutense University of Madrid, Madrid, Spain; 2 Inmunotek, Alcalá de Henares, Madrid, Spain; 3 Fundación Investigación Hospital Clínico San Carlos, Martin Lagos s/n, 28040 Madrid, Spain; 4 Centro de Investigaciones Biológicas Margarita Salas, CSIC, Ramiro de Maetzu 9, 28040 Madrid, Spain; 5 CIBER de Enfermedades Respiratorias (CIBERES) Avda, Monforte de Lemos, 3-5, 28029 Madrid, Spain; 6 Servicio de Urología, Hospital Clínico San Carlos, Universidad Complutense de Madrid, Martín Lagos s/n, 28040 Madrid, Spain

**Keywords:** Tumoral carbohydrate A10, Heparan sulfate-related proteoglycan, Cancer immunology, Dendritic cell, Regulatory T cell, Tumour immunology, Regulatory T cells

## Abstract

Functional Tregs play a key role in tumor development and progression, representing a major barrier to anticancer immunity. The mechanisms by which Tregs are generated in cancer and the influence of the tumor microenvironment on these processes remain incompletely understood. Herein, by using NMR, chemoenzymatic structural assays and a plethora of in vitro and in vivo functional analyses, we demonstrate that the tumoral carbohydrate A10 (Ca10), a cell-surface carbohydrate derived from Ehrlich’s tumor (ET) cells, is a heparan sulfate-related proteoglycan that enhances glycolysis and promotes the development of tolerogenic features in human DCs. Ca10-stimulated human DCs generate highly suppressive Tregs by mechanisms partially dependent on metabolic reprogramming, PD-L1, IL-10, and IDO. Ca10 also reprograms the differentiation of human monocytes into DCs with tolerogenic features. In solid ET-bearing mice, we found positive correlations between Ca10 serum levels, tumor size and splenic Treg numbers. Administration of isolated Ca10 also increases the proportion of splenic Tregs in tumor-free mice. Remarkably, we provide evidence supporting the presence of a circulating human Ca10 counterpart (Ca10H) and show, for the first time, that serum levels of Ca10H are increased in patients suffering from different cancer types compared to healthy individuals. Of note, these levels are higher in prostate cancer patients with bone metastases than in prostate cancer patients without metastases. Collectively, we reveal novel molecular mechanisms by which heparan sulfate-related structures associated with tumor cells promote the generation of functional Tregs in cancer. The discovery of this novel structural-functional relationship may open new avenues of research with important clinical implications in cancer treatment.

## Introduction

Compelling experimental evidence has demonstrated the involvement of the immune system in both tumor rejection and tumor progression [1, 2]. Effective host antitumor responses are challenged by tumor escape mechanisms based on the suppression of effector cells [3–5]. Regulatory T cells (Tregs) are a heterogeneous group of T cells with potent suppressive capacity that are classified as thymus-derived CD25^+^FOXP3^+^ Tregs or peripherally induced Tregs generated outside the thymus upon antigenic stimulation [6]. Tregs are crucial for maintaining tissue homeostasis in many biological contexts, such as autoimmunity, allergies, metabolic syndrome, transplantation, infection, and cancer [1, 3, 4, 7]. Functional Tregs abnormally proliferate in cancer and Treg numbers are greatly increased within tumor tissues, playing a key role in tumor development and progression and posing a major barrier to anticancer immunity [4, 8, 9]. In tumor tissues, Tregs can be generated from local FOXP3^-^ conventional T cells and/or recruited to the tumor site upon previous expansion in lymphoid organs [1, 3, 4, 7]. The mechanisms by which Tregs are generated in hosts bearing malignant tumors and the contribution of the tumor microenvironment (TME) to these processes are not yet fully understood [1, 3, 4, 7]. In this regard, dendritic cells (DCs) are professional antigen-presenting cells that are crucial in the orchestration of proper immune responses by linking innate and adaptive immunity [10]. DCs recognize all surrounding antigens within the tissues, which are then processed and presented to T cells to initiate proper adaptive immune responses depending on the inflammatory context and surrounding environmental cues [10–12]. Under homeostatic noninflammatory conditions, immature DCs remain tolerogenic and generate functional Tregs. The ability of mature DCs to infiltrate tumors and promote the generation of highly suppressive Tregs is strongly influenced by exogenous signals encountered in the TME [10]. However, how DCs produce the resulting functional outcomes from these signals remain unknown.

Malignant transformation is accompanied by changes in tumor cell surface glycoconjugates [13, 14], including the aberrant or incomplete glycosylation of heavily glycosylated structures such as mucins and proteoglycans [15]. These structures are often overexpressed and shed from tumor cells, having a strong influence on tumor progression by modulating cell adhesion, neoangiogenesis and metastasis [16]. In addition, tumor-associated carbohydrates may inhibit anticancer immunity through different mechanisms [17, 18], such as the expansion of myeloid-derived suppressor cells [19] or the activation of Tregs [20]. Whether tumor-derived carbohydrate structures encountered by DCs triggers DC-mediated functional Treg generation, the underlying molecular mechanisms and the potential clinical implications in tumor development, progression and cancer treatments remain incompletely understood.

Murine Ehrlich’s tumor (ET) cells, originally derived from a spontaneous mammary carcinoma, lack H-2 antigens and can grow either in ascitic or solid form in almost any mouse strain across different antigenic backgrounds, representing a suitable model to investigate potential novel tumor escape mechanisms [21]. The carbohydrate A10 (Ca10) is a highly glycosylated ET cell surface macromolecule defined by the binding of monoclonal antibody (mAb) A10 [22, 23]. Ca10 is spontaneously released from ET cells and can be detected in the serum of solid ET-bearing mice [23]. Although the mAb A10 also reacts with glycan moieties located in some human cancer mucins [24], the structural features of the carbohydrate moiety contained in Ca10 and its potential functional implications in cancer remain largely unknown. Herein, we show positive correlations between serum levels of Ca10, tumor size and the generation of splenic Tregs in mice bearing solid ET. The administration of isolated Ca10 to tumor-free mice significantly increases the number of splenic Tregs. We find that Ca10 possesses mainly heparan sulfate-related structures that endow human DCs with the capacity to generate functional Tregs by mechanisms partially dependent on PD-L1, IL-10, IDO, the mTOR pathway and glycolysis. Ca10 also reprograms the differentiation of human monocytes into DCs with tolerogenic features. Additionally, we demonstrate, for the first time, that the levels of a human Ca10 homolog (Ca10H) is increased in the sera of patients suffering from different types of cancer compared to healthy controls and that its serum levels correlate with bone metastases in prostate cancer patients.

## Materials and methods

### Media and reagents

RPMI 1640 (Lonza) supplemented with 10% fetal bovine serum (FBS), 100 μg/mL normocin (InvivoGen), 50 μg/mL penicillin‒streptomycin, 1% nonessential amino acids, 1% MEM vitamins and 1 mM sodium pyruvate was used. AIM V medium (Gibco) was employed for the in vitro expansion of Tregs. Ham’s F12 medium (Lonza) supplemented with 10% FBS was used for PC3 and B16 cell line culture. Inhibitors for IDO (1-methyl tryptophan, 1-MT), glycolysis (2-deoxy-D-glucose, 2-DG), NF-κB (BAY117082) (Sigma‒Aldrich), Syk (piceatannol) (Enzo Life Sciences), MEK 1/2 (U0126), p38 (SB202190), JNK (SP600125), mTOR (rapamycin) (InvivoGen), calcium chelating agent (EDTA), heparin (Laboratorio Reig Jofré) and the corresponding vehicles were used. Blocking antibodies for IL-10 (clone JES3-9D7, Biolegend) and PD-L1 (clone 29E.2A3, Biolegend) and corresponding isotype controls were used for the blocking experiments. We used lipopolysaccharide (LPS) from *Escherichia coli* O127:B8 (Sigma‒Aldrich).

### Ehrlich tumor (ET), PC3 and B16-F10 cell lines

ET cells, initially originating from hyperdiploid Ehrlich-Lettré mouse ascites tumor cells, are derived from a cloned cell variant selected for its high reactivity with a monoclonal A10 antibody. PC3 cells were kindly provided by Dr. Eduardo Martínez-Naves, Department of Immunology, Ophthalmology and ENT, School of Medicine, Complutense University of Madrid, Madrid, Spain. B16-F10 cells were provided by the Department of Immunology, Hospital Clínico San Carlos, Instituto de Medicina del Laboratorio (IML), Complutense University of Madrid, Madrid, Spain.

### Isolation of Ca10 from ET cells

Ca10 was obtained from the supernatants of ET cells growing in vitro in serum-free medium for 24 h. Briefly, the pooled supernatants collected by centrifugation were subjected to tangential ultrafiltration using 300 kDa membranes (Pall Corporation) under high ionic strength (1 M NaCl). The Ca10-enriched fraction obtained from the retentate was subsequently thawed with distilled water on 300 kDa membranes and lyophilized, having a relative composition of 85 ± 5% carbohydrate and 10 ± 5% protein with no significant amounts of nucleic acids or lipids, as observed by NMR. In some experiments, Ca10 was obtained from ET cells cultured for 72 h in the presence or absence (control) of GAG biosynthesis inhibitors: per-O-acetyl-4-fluoro-4-deoxy-GlcNAc (125 μM) (Biosynth-Carbosynth), 4-fluoro-4-deoxy-GlcNAc (1 mM) (Biosynth-Carbosynth), sodium chlorate (30 mM) (Sigma‒Aldrich), and xyloside (1 mM) (4-methylumbelliferyl-beta-D-xylopyranoside; Apollo Scientific).

### Monoclonal A10 antibody

The murine IgM monoclonal A10 antibody (mAb A10) was provided by Inmunotek S.L. and purified from the culture supernatant of a cloned A10-producing hybridoma. A10 was obtained by immunization with devitalized ET cells and selected by their reactivity against carbohydrates on the surface of murine ET cells and their ability to inhibit tumor growth in vivo [22].

### Animal studies

For all of the experiments, the animals were maintained in the Experimental Medicine and Surgery Department, Hospital Clínico San Carlos (Madrid, Spain) under the provisions of R.D. 53/2013 and D.C. 86/609/CEE; RD 1201/2005. C57BL/6J mice (7 weeks old, Charles River) were inoculated with 10^5^ ET cells by subcutaneous injection into the left groin. Tumor size was monitored weekly by calculating the tumor area (mm^2^, width × length) and tumor volume (mm^3^, (4*π*/3) × (width/2)^2^ × (length/2)). The tumor length and width were measured with a Vernier caliper. Mice were sacrificed on different days (22, 32, 42, and 58 days) of tumor development. After sacrifice, taking all tumors into consideration, the average tumor volume was determined to be 246 mm^3^, with mice bearing small (below the average, <246 mm^3^) and large (above the average, >246 mm^3^) tumors. For some experiments, C57BL/6J tumor-free mice were alternately administered (i.v./i.p.) isolated Ca10 (40 μg) in the presence or absence of the mAb A10 (80, 160 or 320 μg) for 7 days, and mice were sacrificed the day after the last inoculation. In all experiments, spleen and blood samples, collected by cardiac puncture, were obtained to quantify splenic Treg numbers and circulating Ca10 levels, respectively, as described below.

### Quantification of Ca10 levels

Ca10 levels were measured in ET cell culture supernatants, mouse and human sera and isolated Ca10 preparations subjected to different treatments by a tailor-made mAb A10-based sandwich ELISA. In this assay, purified mAb A10 (IgMκ) from culture supernatants of the mAb A10-producing hybridoma was used as the capture antibody, and horseradish peroxidase-labeled mAb A10 (HRP-A10) from the same source was used as the detection antibody. Plates were coated overnight at 4 °C with 5 μg/mL mAb A10 in 0.05 M carbonate-bicarbonate buffer (pH 9.4). Then, they were washed with PBS 0.25% Tween-20 (PBS-Tw) and incubated for 2 h at room temperature (RT) with the samples diluted in PBS-Tw. After a wash step, a dilution of HRP-mAb A10 (1:1000 dilution) in PBS-Tw and 5% FBS was added and incubated for 2 h at RT. After a final wash, the peroxidase substrate (OPD, 0.63 mg/mL) (Thermo-Fischer) was added to 0.1 M citrate buffer with 0.03% H_2_0_2_ - pH 5.5. The enzymatic reaction was allowed to develop for 30 min and stopped by adding a 10% HCl solution. The Ca10 concentration was expressed as arbitrary units (AU) per mL by extrapolating the OD values (495 nm) of a reference curve established with Ca10. The detection and quantitation limits were established as 0.03 and 0.05 AU/mL, respectively, with a linear range between 0.03 and 1.56 AU/mL (*R*^2^ > 0,98). Sensitivity and specificity (% of recovery = 99.85) assays were performed to validate its use for Ca10 analysis. In some experiments, the samples to be tested for Ca10 were previously subjected to enzymatic treatment: (i) Bacteroides heparinase II plus heparinase III (New England Biolabs) prepared in heparinase reaction buffer at final concentrations of 85 AU/mL and 14.8 AU/mL, respectively, for 24 h at 30 °C; (ii) *Streptomyces griseus* Pronase (Roche) prepared in 10 mM CaCl_2_ at a final concentration of 0.2 mg/mL for 24 h at 40 °C; and (iii) Sialidase 33 A (Nzytech) prepared in reaction buffer at a final concentration of 0.375 μg/mL. After enzymatic treatment, enzymes were inactivated at 100 °C for 1 min. To monitor oxidized Ca10 (Ca10 OX) reactivity, Ca10 was previously oxidized with sodium periodate (50 mM) (Sigma‒Aldrich) for 70 min at RT in the dark. To remove the reagent, distilled water was added, and the sample was centrifuged for 10 min at 14,000 × *g* using a Nanosep 3 K Omega (Pall Corporation).

### Generation of hmoDCs, purification of naive CD4^+^ T cells and isolation of DCs

Peripheral blood mononuclear cells (PBMCs) from buffy coats of healthy donors were obtained by using Ficoll density gradient centrifugation (800 × *g*, 20 min). Monocytes were isolated from total PBMCs using anti-CD14 microbeads and cultured for 6 days with RPMI medium containing 100 ng/mL IL-4 and GM-CSF (PeproTech) to generate immature human monocyte-derived dendritic cells (hmoDCs). To generate Ca10/hmoDCs, Ca10 (20 μg/mL) was added on Days 0 and 4 of differentiation. Peripheral blood naive CD4^+^ T cells and DCs were isolated using the “Naive CD4^+^ T-Cell Isolation Kit” and “Blood Dendritic Cell Isolation Kit II” (Miltenyi Biotec), respectively, according to the manufacturer’s protocol. In all cases, the purity and phenotype of cells were analyzed by flow cytometry with lineage-specific markers, and cell viability was determined by trypan blue exclusion with a light microscope.

### Human cell cultures

Immature hmoDCs or human total blood DCs(10^6^ cells per mL) were treated with Ca10 (20 µg/mL) for 18 h. Subsequently, the cells were collected and centrifuged. Flow cytometry and qPCR were used to analyzed the cell phenotypes, and ELISA was used to quantify the levels of TNF-α, IL-6, IL-1β and IL-10 in the cell-free supernatants. For inhibition experiments, hmoDCs were preincubated for 30 min with EDTA (0.5 mM), piceatannol (25 μM), U0126 (1 μM), SB202190 (1 μM), SP600125 (10 μM), BAY117082 (2.5 μM), 1-MT (1 mM), rapamycin (100 nM), or heparin (100 IU/ml) and for 1 h with 2-DG (10 mM) or anti-IL-10 (2.5 μg/ml) or the corresponding vehicle control or isotype control prior to treatment. Then, hmoDCs were treated with Ca10 for 18 h in the presence of the corresponding inhibitors. To analyze the contribution of the carbohydrate structure, hmoDCs were stimulated with 20 μg/mL Ca10 (without treatment), oxidized Ca10 (Ca10 OX), Ca10 treated with heparinase (HPSE treat.) or Ca10 treated with pronase (Ca10+Pronase) for 18 h. For blocking experiments with the mAb A10, Ca10 was previously incubated for 1 h with mAb A10 or the corresponding isotype control at a ratio of 1:10 or 1:5 (Ca10:A10) with continuous stirring, and then, it was added to hmoDCs for 18 h. Ca10/hmoDCs or conventional hmoDCs (10^6^ cells per mL) were treated with medium (negative control) or LPS (100 ng/mL) for 18 h. Flow cytometry and qPCR were used to analyzed the cell phenotypes, and ELISA was used to quantify the levels of TNF-α, IL-6, IL-1β and IL-10 in the cell-free supernatants. In all cases, cell viability was analyzed by trypan blue exclusion under microscopy.

### Migration (wound healing) and proliferation (MTT) assays

PC3 and B16-F10 cells were detached from the tissue culture plate using TripLE^TM^ Express (Gibco). Cells were centrifuged and resuspended in culture media. Cells were plated in a 48-well plate for 100% confluence in 24 h. A wound approximately 100 µm wide was generated using a pipette tip to measure wound healing (cell migration). Media and cell debris were removed, and culture media were added with or without Ca10 (20 µg/mL) for 48 h. The width of the scratch was measured at different time points and represented relative wound closure. Migration was recorded at 6 time points (0, 4, 8, 12, 24 and 48 h) using optical microscopy.

PC3 and B16-F10 cells were seeded at 5 × 10^4^ cells per well in a 96-well plate for 24 h. Then, media and cell debris were removed, and culture media were added with or without Ca10 (20 µg/mL) for 48 h. Cell proliferation at 24 and 48 h was analyzed following the Cell Proliferation Kit MTT standard protocol (Roche).

### Flow cytometry

The following anti-human mAbs were used for flow cytometry: fluorescein isothiocyanate (FITC)-conjugated anti-HLA-DR; phycoerythrin (PE)-conjugated anti-CD86; allophycocyanin (APC)-conjugated anti-HLA-DR; FITC-conjugated anti-CD1c; and PE-conjugated anti-CD303 (Miltenyi Biotec); APC-conjugated anti-CD83; FITC-conjugated anti-PD-L1; phycoerythrin-Cy7 (PE/Cy7)-conjugated anti-CD19; peridinin-chlorophyll-protein (PerCP)-conjugated anti-CD4; Alexa Fluor 488-conjugated anti-FOXP3; PE-conjugated anti-CD127; and APC-conjugated anti-CD25 (BioLegend); and PE-conjugated anti-ICOSL (BD Pharmingen). Cells were washed with PBS 2 mM EDTA and 0.5% BSA and stained for 15 min at RT in the dark. For analysis of FOXP3 expression in human T cells cocultured with DCs, the cells were first subjected to surface staining with anti-human PE-CD127, PerCP-CD4, and APC-CD25 antibodies. After fixation and permeabilization, the cells were stained with Alexa Fluor 488-FOXP3 according to the manufacturer’s recommendations. The same protocol was carried out for the characterization of CD4^+^CD25^high^FOXP3^+^ Tregs in mouse splenocytes using the following anti-mouse mAbs: PerCP-conjugated anti-CD4, PE-conjugated anti-CD25, and Alexa Fluor 488-conjugated anti-FOXP3 (BioLegend). The viability dye eFluor 660 (eBioscience) was used to assess viability by flow cytometry, and dead cells were excluded from the analysis. For each staining, the corresponding isotype controls (IgG2A-FITC, IgG1-Alexa Fluor 488, IgG1-PE, IgG2A-PerCP, or IgG1-APC) were also assayed. The following antibodies were used for the flow cytometry analysis of Ehrlich tumor cells: A10 and anti-mouse IgM (μ-chain specific)-FITC produced in goat (Sigma‒Aldrich). Flow cytometry analysis was performed using a FACSCalibur in the Cytometry and Fluorescence Microscopy Unit at Complutense University of Madrid.

### Cytokine quantification

The levels of TNF-α, IL-6, IL-1β, IL-10, IFN-γ and IL-5 in cell-free supernatants were quantified by sandwich ELISA kits (BD Biosciences) following the manufacturer’s instructions.

### RNA isolation, cDNA synthesis, and quantitative real-time RT‒PCR

RNA was isolated from harvested cells using an RNeasy mini kit (Qiagen), and cDNA was generated using a PrimeScript RT reagent Kit (Takara) according to the manufacturers’ instructions. Real-time quantitative PCR was performed on cDNA using FastStart Universal SYBR Green Master (Rox) (Roche). The sequences of the primer pairs used were as follows: *IL6* (forward, GGTACATCCTCGACGGCATCT; reverse GTGCCTCTTTGCTGCTTTCAC), *IL1B* (forward, TTTTTGCTGTGAGTCCCGGAG; reverse TTCGACACATGGGATAACGAGG), *IL10* (forward, GTGATGCCCCAAGCTGAGA; reverse CACGGCCTTGCTCTTGTTTT), *PDL1* (forward, AAGATGAGGATATTTGCTGTCTTTATATTC; reverse, GTCCTTGGGAACCGTGACAGT), *indoleamine 2,3-dioxygenase* (*IDO*) (forward, AGAAGTGGGCTTTGCTCTGC; reverse, TGGCAAGACCTTACGGACATCTC), *suppressor of cytokine signaling 1* (*SOCS1*) (forward, CCCTGGTTGTTGTAGCAGCTT; reverse, CAACCCCTGGTTTGTGCAA), *SOCS3* (forward, CCTCAGCATCTCTGTCGGAAGA; reverse, GCATCGTACTGGTCCAGGAACT), *retinaldehyde dehydrogenase 1 (RALDH1)* (forward, CTGCCGGGAAAAGCAATCT; reverse, AAATTCAACAGCATTGTCCAAGTC), *RALDH2* (forward, AGGGCAGTTCTTGCAACCAT; reverse, GCGTAATATCGAAAGGT). Samples were run on a real-time PCR system (ABI Prism 7900 HT; Applied Biosystems). Data were normalized to *EF1A* and displayed as arbitrary units calculated as 2^−ΔCT^ values multiplied by 10^4^. ΔCT was defined as the difference between the cycle threshold value for the gene of interest and *EF1A*.

### Western blot analysis

HmoDCs (10^6^ cells per mL) were treated with Ca10 for 30 min at 37 °C. Then, cells were harvested and lysed with RIPA buffer (Thermo Fisher Scientific) in the presence of protease/phosphatase inhibitor cocktail (Cell Signaling) for 30 min at 4 °C with vortexing every 10 min. Lysates were clarified by centrifugation at 10,000 × *g* for 15 min at 4 °C. Protein quantification was performed with a Micro BCA Protein Assay Kit (Pierce), and samples with equal amounts of total protein were resolved by 10% SDS-polyacrylamide gel electrophoresis (SDS–PAGE). Proteins were then transferred to nitrocellulose membranes (Bio-Rad). The membrane was incubated with the following primary antibodies: phospho-ERK1/2 (Thr202/Tyr204), ERK1/2, phospho-SAPK/JNK (Thr183/Tyr185), SAPK/JNK, phospho-p38 MAPK (Thr180/Tyr182), p38 MAPK, phospho-IκBα (Ser32/35) or IκBα (1:1000, Cell Signaling), and β-actin (1:15000, Sigma‒Aldrich) and goat anti-rabbit (1:4000, Bio-Rad) or goat anti-mouse (1:2500, Pierce) conjugated with horseradish peroxidase as a secondary antibody. The signals were visualized with Clarity Western ECL Substrate (Bio-Rad) and detected in a Fujifilm LAS-3000 developer.

### Metabolic studies

HmoDCs were stimulated with Ca10 for 18 h. For real-time metabolic characterization, the extracellular acidification rate (ECAR, mpH/min), mitochondrial oxygen consumption rate (OCR, pmol/min), and glycolytic proton efflux rate (glycoPER, pmol/min) were analyzed using a Seahorse XF HS Mini Analyzer (Agilent). HmoDCs after Ca10 stimulation and fresh Ca10/hmoDCs were harvested, washed and resuspended in DMEM supplemented with 10 mM glucose, 1 mM pyruvate and 2 mM glutamine. Cells (25 × 10^3^/well) were plated in poly-D-lysine-coated 8-well miniplates and incubated in a non-CO_2_ incubator for 1 h at 37 °C. ECAR and OCR were analyzed using a glycolysis rate assay with final concentrations of 1 μM rotenone, 1 μM antimycin A, and 50 mM 2-deoxyglucose (2-DG). A complete glycoPER study was performed in two consecutive stages: basal glycolysis (without drugs) and electron transportation chain inhibition (rotenone and antimycin A). The OCR was analyzed using a Cell Mito Stress Test with final concentrations of 1.5 μM oligomycin, 1 μM carbonyl cyanide 4-(trifluoromethoxy) phenylhydrazone (FCCP), 0.5 μM rotenone and 0.5 μM antimycin A. A complete OCR study was performed in four consecutive stages: basal respiration (without drugs), mitochondrial complex V inhibition (oligomycin), maximal respiration induction (FCCP), and electron transportation chain inhibition (rotenone and antimycin A). The glucose concentration in cell-free supernatants was determined by using the Glucose (GO) Assay Kit (Sigma‒Aldrich). The metabolic rate was calculated as the percentage of the medium without hmoDCs (2 mg/mL). The Warburg effect was determined by quantifying the optical density (OD) at 570 nm and calculated as 1/OD570. Lactate production in cell-free supernatants was determined by using a colorimetric L-lactate assay kit (Sigma‒Aldrich).

### Coculture experiments and Treg suppression assay

Immature hmoDCs or total DCs were stimulated with Ca10 for 18 h as well as Ca10/hmoDCs and conventionally treated with medium for 18 h. Next, they were washed and cocultured with purified allogeneic naive CD4^+^ T cells (1:5 DC/T cells) for 5 days. The levels of IFN-γ, IL-5 and IL-10 were quantified in cell-free supernatants by ELISA, and the expression of FOXP3 was analyzed by flow cytometry. Inhibition assays were performed by adding anti-human IL-10 (2.5 μg/mL) or anti-human PD-L1 (10 μg/mL) to coculture. The induced CD4^+^CD127^-^CD25^high^FOXP3^+^ Tregs by allogeneic Ca10-treated hmoDCs were purified by sorting CD4^+^CD127^-^CD25^high^ cells and mixed with carboxyfluorescein succinimidyl ester (CFSE)-labeled autologous PBMCs (responder cells) at different ratios and stimulated with plate-bound anti-human CD3 antibody (1 μg/mL, clone OKT3; eBioscience) and soluble anti-human CD28 (1 μg/mL, clone CD28.6; eBioscience) for 5 days. For control purposes, CFSE-labeled PBMCs were cultured alone with or without stimulation, and CD4^+^CD127^+^CD25^-^ cells (non-Tregs) were also mixed with CFSE-labeled PBMCs. The proliferation of CD4^+^ T cells was determined by CFSE dilution and flow cytometry.

### Differentiation and expansion of human Tregs

For the in vitro generation of Tregs, peripheral blood naive CD4^+^ T cells were isolated using the “Naïve CD4^+^ T-Cell Isolation Kit” (Miltenyi Biotec). Then, the cells (10^6^ cells per mL) were stimulated in 48-well plates in AIM V medium at 37 °C with 100 U/mL IL-2, 10 ng/mL TGF-β (PeproTech), 25 µL/mL ImmunoCult™ Human CD3/CD28/CD2 (CDmix) T-Cell Activator (STEMCELL Technologies), 5 µg/mL anti-IL-12/IL-23, 5 µg/mL anti-IL-4 and 5 µg/mL anti-IFN-γ (Biolegend) in the presence or absence of Ca10 (20 µg/mL). On Day 2, cell cultures were supplemented with 100 U/ml IL-2 in cRPMI medium. On Day 4, the cells were split, and the cultures were supplemented with 100 U/ml IL-2 in cRPMI medium. On Day 5, the cells were collected and centrifuged. For expansion experiments, the obtained Tregs (10^6^ cells per mL) in the absence of Ca10 were restimulated in 48-well plates in cRPMI medium at 37 °C with 100 U/mL IL-2 and 25 µL/mL ImmunoCult™ Human CD3/CD28/CD2 (CDmix) T-Cell Activator in the presence or absence of Ca10 (20 µg/mL). On Day 1, cell cultures were supplemented with 100 U/ml IL-2 in cRPMI medium. On Day 2, the cells were split, and the cultures were supplemented with 100 U/ml IL-2 in cRPMI medium every one or two days. On Day 7, the cells were collected and centrifuged. For suppression assays, generated CD4^+^CD25^+^CD127^-^FOXP3^+^ Tregs were mixed with CFSE-labeled autologous PBMCs (responder cells) at different ratios and stimulated with 1 μg/mL anti-CD3, 1 μg/mL anti-CD28 mAb and 100 U/mL IL-2 for 5 days as described above.

### NMR experiments

All NMR spectra were acquired using a Bruker AVANCE 600 MHz spectrometer equipped with a triple resonance TXI cryogenic probe and processed with TOPSIN 3.0 software (Bruker SA). NMR samples were prepared in deuterium oxide (D_2_O). One-dimensional proton (1D–^1^H) spectra, two-dimensional (2D) *diffusion-ordered spectroscopy* (DOSY), 2D heteronuclear ^1^H–^13^C single quantum coherence (^1^H–^13^C HSQC) and 2D homonuclear ^1^H–^1^H *total correlation spectroscopy* (TOCSY) spectra were acquired using standard pulse sequences included in TOPSPIN software to characterize the structure (TOCSY and HSQC) and hydrodynamic behavior (DOSY) of Ca10 samples.

To monitor periodate oxidation, Ca10 was dissolved in 50 mM sodium periodate (NaIO_4_) in D_2_O at a final concentration of 5 mg/mL in an NMR tube and introduced into the spectrometer probe adjusted at 298 K. One-dimensional 1D–^1^H and 2D DOSY spectra were acquired at sequential times to follow the oxidation reaction over 8 h. DOSY spectra were obtained using the standard Bruker pulse sequence (ledbpgp2s), acquiring 16 gradient points, with 128 scans each, between 2 and 95% gradient intensity using a diffusion time delay of 0.25 s and 2500 μs to achieve a wide pulse gradient.

To monitor heparinase digestion, 5 μL of Bacteroides heparinases II and III (400 UA/mL and 700 UA/mL, respectively) were added to the NMR tube containing 5 mg/mL Ca10 sample in heparinase buffer, and the tube was introduced in the spectrometer probe adjusted at 303 K. To evaluate the enzymatic reaction, 1D-^1^H and 2D DOSY spectra were acquired at sequential times for 24 h. The 1D-^1^H spectra of 128 scans of 32 K size were recorded by applying the TOPSIN zgesgp pulse program, which includes a gradient sculpting water suppression procedure. The 2D DOSY experiments were performed using the pulse sequence ledbpgp2s acquiring 32 gradient points, with 128 scans each between 2 and 95% gradient intensity. The diffusion time delay and gradient duration were 600 ms and 2500 μs, respectively, before the start of the reaction and 170 ms and 1700 μs at the end point of the reaction.

To monitor pronase digestion, Ca10 dissolved in deuterated buffer at 5 mg/mL was treated with pronase as indicated above. DOSY spectra were acquired at 298 K before and after pronase treatment with the same parameters used for the periodate oxidation sample.

#### Enzyme digestions

A collection of glycosidases was obtained: N-glycosidase (PNGase F), O-glycanase (endo-galactosaminidase), endo-β-acetylglucosaminidase, exoglycosidases (α- and β- galactosidases), α-mannosidase, glucosidases, glucosaminidase, α-fucosidase, sialidase (NZYTECH), chitosanase 8B, heparinases (New England Biolabs) and chondroitinase ABC. Working conditions with each enzyme were determined using model glycoproteins such as α1-acid glycoprotein, fetuin, asialofetuin, ribonuclease B and commercial oligosaccharides. Enzymatic digestions were analyzed by NMR, polyacrylamide gel electrophoresis (SDS‒PAGE) and Ca10-epitope sandwich ELISA evaluation.

### Heparinase products and commercial heparan sulfate disaccharides

The sample of heparinase-digested Ca10 was filtered using Vivaspin™ 50 K Centrifugal Concentrators (Sartorius), and the filtrate was lyophilized and resuspended in D_2_O for NMR analysis. 1D–^1^H, 2D TOCSY, ^1^H–^13^C HSQC and DOSY spectra were acquired. For comparison, a collection of heparan sulfate disaccharide standards were also analyzed by NMR: ∆UA,2S – GlcNAc,6S (I-A) ∆UA – GlcNS,6S (II-S), ∆UA – GlcNS (IV-S), ∆UA – GlcNAc (IV-A), ∆UA,2S – GlcNAc (III-A) (Iduron, UK) and ∆UA – GlcN (IV-H) (Santa Cruz Biotechnology). Samples of each of them at a 1 mM concentration in D_2_O were prepared, and 1D–^1^H, 2D ^1^H–^13^C HSQC and DOSY spectra were acquired for comparison.

### Human serum samples

Sera from patients diagnosed with prostate adenocarcinoma (*n* = 248), colorectal adenocarcinoma (*n* = 66) and other types of cancer (*n* = 71) at any stage of the disease were obtained from the Central Laboratory of Hospital Clínico San Carlos (Madrid, Spain) during routine follow-up. The levels of Ca10H in the serum samples of prostate cancer patients with (*n* = 42) or without (*n* = 30) bone metastases from the Urology Department were measured, as described above for Ca10, and alkaline phosphatase levels were measured by spectrophotometry (AU5800 Clinical Chemistry Analyzer, Beckman Coulter). The serum samples from the healthy donors (*n* = 131) used as controls were obtained from the Blood Donor Unit of the Hospital Clínico San Carlos.

### Ethics approval

All patients and controls signed informed consent documents, and the data were treated according to recommended criteria of confidentiality, following the ethical guidelines of the 1975 Declaration of Helsinki. The study was approved by the local Ethics Committee (Hospital Clínico San Carlos, Madrid, CEIM 13/098).

### Statistics

All the data are expressed as the mean ± SEM of the indicated parameters. Spearman’s correlation, paired or unpaired Student’s *t* test, the Wilcoxon test and the Mann‒Whitney *U* test were used for statistical analysis performed by GraphPad Prism software, version 8.0. Significance was defined as *P < 0.05, **P < 0.01, ***P < 0.001 and ****P < 0.0001.

## Results

### Serum levels of Ca10 positively correlate with tumor size and splenic Treg numbers in ET-bearing mice

**Fig. 1:**
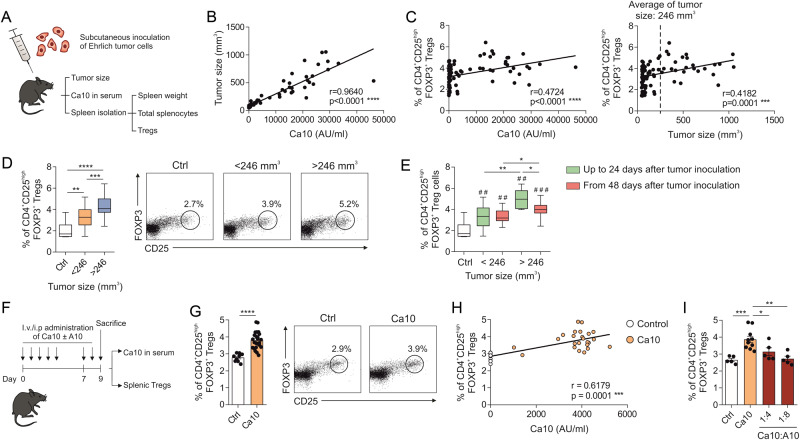
Increased percentages of Tregs in mice bearing Ehrlich tumors (ETs). **A** Protocol of ET cell subcutaneous inoculation in mice. Correlation of serum Ca10 levels with tumor size (mm^3^) (**B**) and percentage of splenic Tregs with serum Ca10 levels or tumor size (mm^3^) (**C**) in ET-bearing mice (*n* = 79 mice of two independent experiments). **D** Percentage of splenic Tregs from no tumor-bearing mice (ctrl) and mice with small (<246 mm^3^) or large (>246 mm^3^) tumors. The average tumor size was used as the cutoff (246 mm^3^). Representative dot plots of Tregs are shown (*n* = 6 for ctrl, *n* = 48 and 25 for small and large tumors, respectively, of two independent experiments). **E** Percentage of splenic CD4^+^CD25^high^FOXP3^+^ Tregs from no tumor^-^bearing mice (ctrl) and small or large tumor-bearing mice. Mice were sacrificed up to 24 days after tumor inoculation (green) or from 48 days after tumor inoculation (red) (*n* = 6 mice for ctrl, *n* = 30 mice with small tumors sacrificed up to 24 days after ET inoculation, *n* = 18 mice with small tumors sacrificed from 48 days after ET inoculation, *n* = 6 mice with large tumors sacrificed up to 24 days after ET inoculation, and *n* = 19 mice with large tumors sacrificed from 48 days after ET inoculation of two independent experiments). # tumor-bearing mice vs. control, * tumor condition vs. tumor condition. **F** Scheme of the alternative intravenous (i.v.) and intraperitoneal (i.p.) administration protocol of Ca10 in the presence or absence of the mAb A10 in mice. **G** Percentage of splenic CD4^+^CD25^high^FOXP3^+^ Tregs from control mice and mice treated with Ca10. Representative dot plots of generated Tregs are shown (*n* = 9 mice for ctrl and *n* = 24 mice for Ca10, of two independent experiments). **H** Correlation of the percentage of splenic Tregs with serum Ca10 levels in control mice (*n* = 9) and Ca10-administered mice (*n* = 24 mice of two independent experiments). **I** Percentage of splen**i**c CD4^+^CD25^high^FOXP3^+^ Tregs from control mice and mice treated with Ca10 in the absence or presence of the mAb A10 (*n* = 5 mice for ctrl, *n* = 10 mice for Ca10 and *n* = 4 mice for Ca10 in the presence of mAb A10 at ratios 1:4 or 1:8, of one independent experiment). Values are mean ± SEM. Statistical significance was determined using the Spearman test (**B**, **C**, **H**), Mann‒Whitney test (**D**, **E**) and unpaired Student’s *t* test (**G**, **I**). *P < 0.05, **P < 0.01, ***P < 0.001, and ****P < 0.0001

ET is a paradigmatic example of tumor progression across different antigenic backgrounds, representing a suitable model to study tumor escape mechanisms. To assess the potential relationship between circulating Ca10 levels, tumor size and Treg generation, we used solid ET-bearing mice (Fig. 1A). A strong positive correlation was observed between serum Ca10 levels and tumor size (Fig. 1B). The proportion of splenic FOXP3^+^ Tregs also showed a moderate but significant positive correlation with both serum Ca10 levels and tumor size (Fig. 1C). Consistent with previous studies, strong splenomegaly related to tumor size was concomitantly observed with a positive correlation between the total number of splenocytes and tumor size (Supplementary Fig. 1a, b). As a result of the tumor size variability among the different ET-bearing mice, cutoff point of 246 mm^3^ (the average tumor size) was set to stratify mice bearing large (>246 mm^3^) and small (<246 mm^3^) tumors. The percentage of splenic FOXP3^+^ Tregs was significantly higher in mice bearing large tumors than in mice bearing small tumors, regardless of the time elapsed since tumor inoculation, and always higher than that in control mice (Fig. 1D). The percentage of splenic Tregs in mice bearing large tumors (>246 mm^3^) that developed at earlier time points (up to 24 days after tumor inoculation) was significantly higher than that in large tumors reaching that size at later time points (from 48 days onward after inoculation of ET cells) (Fig. 1E). These data suggest a close relationship between serum Ca10 levels, the induction of splenic Tregs, and tumor progression. To further investigate whether Ca10 might be directly involved in the observed increase in Treg numbers, isolated Ca10 was administered to C57BL/6J tumor-free mice either alone or in combination with the mAb A10 (Fig. 1F). Remarkably, the percentage of splenic FOXP3^+^ Tregs was significantly higher in mice administered Ca10 than in control mice, which was observed just after 9 days of Ca10 administration (Fig. 1G). Of note, in these experiments, a significant positive correlation between serum Ca10 levels and the percentage of splenic Tregs was observed (Fig. 1H). The administration of Ca10 in combination with the mAb A10 significantly impaired the generation of splenic Tregs induced by the administration of Ca10 in a dose-dependent manner, indicating that mAb A10 completely blocks the Treg induction capacity of Ca10 (Fig. 1I). Collectively, these results suggest that Ca10 shedding by ETs might promote the generation of Tregs, which in turn might favor tumor progression.

### Ca10 endows human DCs with tolerogenic features

**Fig. 2:**
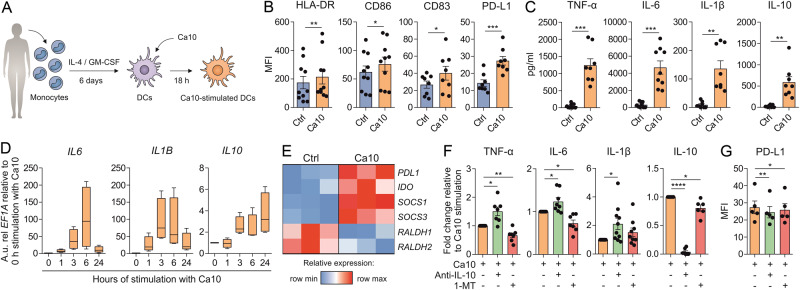
Ca10 induces tolerogenic human DCs. **A** Human in vitro model for the generation of human monocyte-derived DCs. **B** Mean fluorescence intensity (MFI) values for the surface markers HLA-DR, CD86, CD83 and PD-L1 after stimulation of human DCs with medium (Ctrl) or Ca10 for 18 h (*n* = 8–10). **C** Cytokine production after stimulation of human DCs with medium or Ca10 for 18 h quantified in cell-free supernatants by ELISA (*n* = 8–9). **D** Messenger RNA expression levels of the *IL6*, *IL1B* and *IL10* genes in human DCs treated with Ca10 for 0, 1, 3, 6 and 24 h relative to Ca10 stimulation at 0 h. Arbitrary units (A.u.) are 2^−ΔCT^ values multiplied by 10^4^, with ΔCT defined as the difference between the cycle threshold value for the gene of interest and *EF1A* (*n* = 4). **E** Heatmap representation of gene expression in human DCs unstimulated or stimulated with Ca10 for 24 h (*n* = 3). **F** Cytokine production of human DCs stimulated with Ca10 in the presence of IL-10 blocking antibody or IDO inhibitor (1-MT) for 18 h relative to Ca10 stimulation (*n* = 6-10). **G** MFI values for the expression of the surface marker PD-L1 on human DCs after stimulation with Ca10 in the presence of IL-10 blocking antibody or 1-MT for 18 h (*n* = 5). Values are mean ± SEM. Statistical significance was determined using paired Student’s *t* test. *P < 0.05, **P < 0.01, ***P < 0.001, and ****P < 0.0001

To further explore the mechanisms involved in the potential capacity of Ca10 to promote Treg generation, we initially investigated the ability of this tumor-associated carbohydrate to regulate the phenotype and function of human DCs. Ca10 stimulation of DCs differentiated from the human blood monocytes of healthy donors (Fig. 2A) significantly upregulated the expression of HLA-DR, CD86/CD83 and the tolerogenic marker PD-L1 (Fig. 2B). Ca10-stimulated DCs also produced significantly higher levels of TNF-α, IL-6, IL-1β and the anti-inflammatory cytokine IL-10 than unstimulated cells (Fig. 2C). Kinetic experiments showed that Ca10 rapidly increased the mRNA levels of proinflammatory cytokines in a time-dependent manner, which declined to almost basal levels after 24 h of stimulation (Fig. 2D). In contrast, the mRNA levels of IL-10 (Fig. 2D) and the tolerogenic molecules PD-L1, IDO, SOCS1 and SOCS3 (Fig. 2E) remained elevated after 24 h of stimulation, suggesting that Ca10 might endow human DCs with tolerogenic features [25]. Supporting these data, blocking IL-10 significantly enhanced the production of the proinflammatory cytokines TNF-α, IL-6 and IL-1β, whereas treatment with 1-methyl tryptophan (1-MT), a selective inhibitor of IDO activity, significantly reduced the production of IL-10 in Ca10-stimulated DCs (Fig. 2F). Blocking IL-10 or IDO also reduced PD-L1 levels in Ca10-stimulated DCs (Fig. 2G), indicating that the tolerogenic features in human DCs endowed by Ca10 are partially dependent on IL-10 and IDO induction.

### Ca10 activates immune pathways and enhances glycolysis in human DCs

**Fig. 3:**
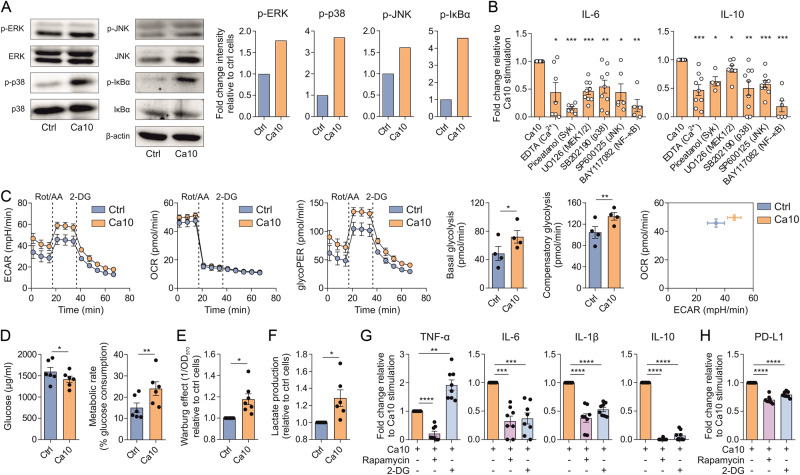
Ca10 induces MAPK and NF-κB signaling pathways and metabolic reprogramming in human DCs. **A** Western blot analysis of protein extracts from human DCs stimulated with or without Ca10 for 30 min. Quantification of the reactive phosphorylated bands by scanning densitometry. β-actin was used as a loading control (*n* = 2–4). **B** Cytokine production of human DCs stimulated with Ca10 for 18 h in the presence of the indicated inhibitors relative to Ca10 stimulation (*n* = 4–9). **C** Kinetic study of the extracellular acidification rate (ECAR), mitochondrial oxygen consumption rate (OCR), glycolytic proton efflux rate (glycoPER) and energy map in Ctrl- or Ca10-stimulated human DCs by sequential addition of rotenone/antimycin A (Rot/AA) and 2-deoxyglucose (2-DG). Quantification of basal glycolysis and compensatory glycolysis in human DCs is included (*n* = 4 of two independent experiments). **D** Glucose consumption by human DCs treated with medium (ctrl) or Ca10 for 18 h and calculated metabolic rate (percentage of glucose consumption) (*n* = 6). **E** Quantification of the induced Warburg effect relative to control cells (*n* = 6). **F** Increase in lactate production in cell-free supernatants from Ca10-stimulated human DCs relative to unstimulated cells (*n* = 6). **G** Cytokine production of DCs stimulated with Ca10 in the presence of an mTOR inhibitor (rapamycin) or glycolysis inhibitor (2-DG) for 18 h relative to Ca10 stimulation (*n* = 8). **H** Mean fluorescence intensity (MFI) values for the surface marker PD-L1 on DCs after stimulation with Ca10 in the presence of rapamycin or 2-DG for 18 h relative to Ca10 stimulation (*n* = 8). Values are mean ± SEM. Statistical significance was determined using paired Student’s *t* test. *P < 0.05, **P < 0.01, ***P < 0.001, and ****P < 0.0001

To gain further insights into the molecular mechanisms involved in the mode of action of Ca10 in human DCs, we assessed its capacity to activate classical immune pathways and to promote metabolic reporgramming. Ca10 induced the rapid activation of mitogen-activated protein kinases (MAPKs) (extracellular-signal regulated kinase (ERK), p38 and c-Jun N-terminal kinase (JNK)) and nuclear factor-κB (NF-κB), as determined by the increase in the phosphorylated forms of the assayed MAPKs and IκBα, an inhibitor of NF-κB (Fig. 3A). No changes in the total levels of ERK, p38 and IκBα were detected, and only an increase in the total level of JNK was observed (Fig. 3A). Supporting these data, the pharmacological inhibitors U0126 (specific for MEK 1/2, upstream of ERK), SB202190 (specific for p38), SP600125 (specific for JNK) and BAY117082 (specific for NF-κB) significantly inhibited IL-6 and IL-10 production in Ca10-activated human DCs (Fig. 3B). IL-6 and IL-10 production induced by Ca10 stimulation was significantly impaired when DCs were preincubated with EDTA (chelating agent of calcium) and piceatannol (spleen tyrosine kinase (Syk) inhibitor) (Fig. 3B). Our inhibition data suggest that even though Ca10 might target other receptors in human DCs, such as Syk-coupled C-type lectin receptors (CLRs), the activation of MAPKs and NF-κB seems to be the main driver of the induced cytokine signature.

Next, we studied the capacity of Ca10 to promote metabolic reprogramming in human DCs. We performed real-time extracellular acidification rate (ECAR), mitochondrial oxygen consumption rate (OCR), and glycolytic proton efflux rate (glycoPER) dynamic experiments using a Seahorse bioanalyzer. Basal glycolysis and compensatory glycolysis were significantly increased in Ca10-stimulated human DCs compared to unstimulated cells (Fig. 3C). We did not observe significant changes in basal respiration, maximal respiration, ATP production-coupled respiration or spare respiratory capacity between Ca10-stimulated DCs and unstimulated DCs (Supplementary Fig. 2a). Supporting these data, Ca10 significantly increased the consumption of glucose from the culture medium and the metabolic rate of DCs compared to unstimulated cells (Fig. 3D). Accordingly, Ca10-activated human DCs showed a significantly higher Warburg effect and lactate production in cell culture supernatants than control cells (Fig. 3E, F). Collectively, the data indicate that Ca10 enhances glycolysis and lactic fermentation without affecting mitochondrial oxidative phosphorylation in human DCs. To further analyze the contribution of the mTOR-mediated signaling pathway and glycolysis to the tolerogenic properties exerted by Ca10 in human DCs, inhibition experiments were performed. The inhibition of the mTOR pathway with rapamycin and glycolysis with 2-deoxyglucose (2-DG) in Ca10-activated DCs significantly suppressed the production of the proinflammatory cytokines TNF-α, IL-6 and IL-1β as well as IL-10, whereas a significant increase in the production of TNF-α was observed in the presence of 2-DG (Fig. 3G). Accordingly, inhibition of mTOR and glycolysis significantly reduced the expression level of PD-L1 induced by Ca10 in human DCs (Fig. 3H), indicating that mTOR-mediated signaling pathways and glycolytic metabolism contribute to the tolerogenic properties of human DCs endowed by Ca10.

### Ca10-stimulated tolerogenic human DCs generate functional Tregs by mechanisms partially dependent on metabolic reprogramming, PD-L1, IL-10 and IDO

**Fig. 4:**
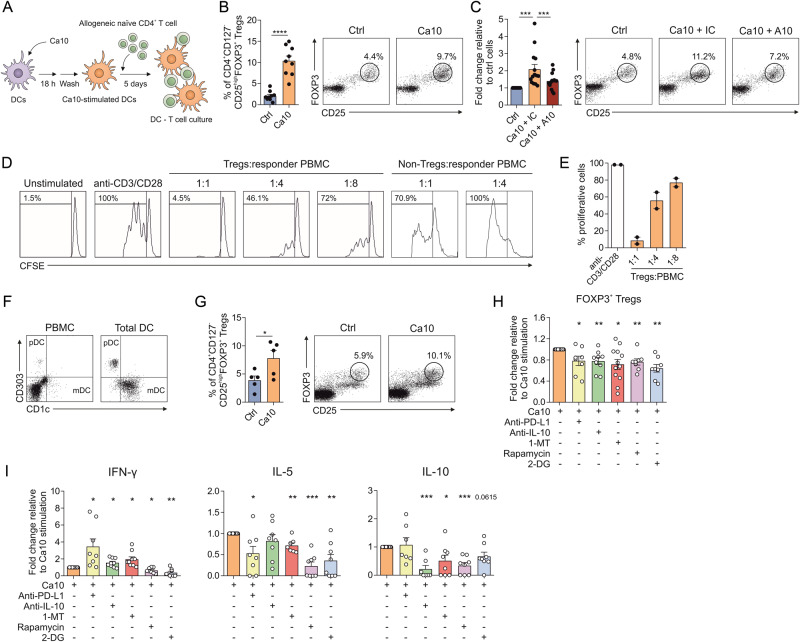
Tolerogenic human DCs induced by Ca10 promote the generation of functional regulatory T cells. **A** Human in vitro model for allogeneic coculture. **B** Percentage of Tregs gated on CD4^+^ T cells after 5 days of coculture with unstimulated or Ca10-treated human DCs (*n* = 9). Representative dot plots are shown. **C** Fold change of Tregs gated on CD4^+^ T cells generated by DCs treated with Ca10 in the presence of the mAb A10 (A10) or the corresponding isotype control (IC) after 5 days relative to control DCs (pool of 1:10 and 1:5 ratios, Ca10:A10) (*n* = 14). **D** Suppression assays of purified Tregs and non-Tregs induced by Ca10-activated DCs. **E** Percentage of proliferating CD4^+^ cells after 5 days of stimulation in the presence of autologous purified Tregs induced by Ca10-activated DCs at different ratios (*n* = 2). **F** Representative dot plots of different DC subsets in PBMCs and total DC fractions gating on HLA-DR^+^CD19^-^ cells. The total DC fraction containing both HLA-DR^+^CD1c^+^ myeloid and HLA-DR^+^CD303^+^ plasmacytoid DCs is shown. **G** Percentage of Tregs by untreated (ctrl) or Ca10-treated total blood DCs after 5 days (*n* = 5). Representative dot plots are shown. **H** Percentage of Tregs induced by Ca10-treated DCs in the presence of blocking antibodies against PD-L1 or IL-10 or inhibitors of IDO (1-MT), mTOR (rapamycin) or glycolysis (2-DG) after 5 days (*n* = 8–12) relative to Ca10 stimulation alone. **I** Cytokines produced by allogeneic naïve CD4^+^ T cells primed by Ca10-activated DCs after 5 days under the indicated conditions relative to Ca10 stimulation alone (*n* = 8–12). Values are mean ± SEM. Statistical significance was determined using paired Student’s *t* test (**B**, **G**, **H**, **I**) and Wilcoxon test I. *P < 0.05, **P < 0.01, ***P < 0.001, and ****P < 0.0001

To determine whether Ca10 could indeed generate human tolerogenic DCs with the capacity to polarize functional Tregs, we performed coculture experiments with allogeneic naïve CD4^+^ T cells (Fig. 4A). Ca10-stimulated human DCs generated a significantly higher percentage of CD4^+^CD127^-^CD25^high^FOXP3^+^ Tregs than unstimulated control DCs (Fig. 4B). When Ca10 was previously incubated with the blocking mAb A10, the number of Tregs induced by Ca10-activated DCs was significantly decreased (Fig. 4C). Remarkably, the Tregs induced by Ca10-stimulated DCs were functional, as they were able to potently suppress, in contrast to the non-Treg fraction, the proliferation of autologous PBMCs in a dose-dependent manner (Fig. 4D, E). Supporting these findings, an enriched fraction of human blood total DCs containing both plasmacytoid (pDC) and myeloid (mDC) DCs (Fig. 4F) stimulated with Ca10 also generated a significantly higher percentage of CD4^+^CD127^−^CD25^high^FOXP3^+^ Tregs than unstimulated total DCs (Fig. 4G). Blocking PD-L1, IL-10, IDO, mTOR and glycolysis significantly impaired the generation of Tregs by Ca10-stimulated DCs (Fig. 4H), which was accompanied by significantly lower levels of IL-5 and IL-10 (Fig. 4I). Blocking PD-L1, IL-10 and IDO induced significantly higher levels of IFN-γ, especially after PD-L1 inhibition, whereas inhibition of mTOR and glycolysis significantly reduced the production of IFN-γ (Fig. 4I).

### Human monocytes differentiated into DCs in the presence of Ca10 generate tolerogenic DCs with the ability to promote Tregs

**Fig. 5:**
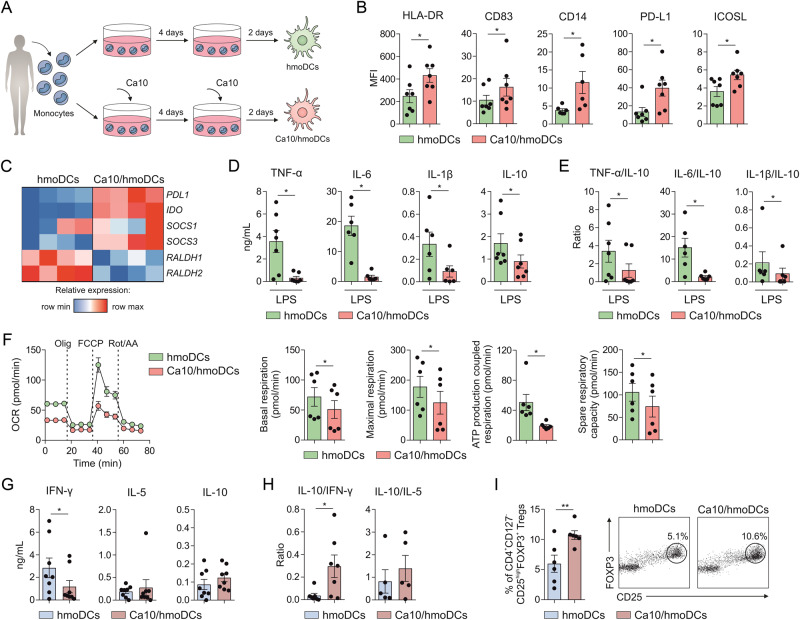
Monocytes differentiated into DCs in the presence of Ca10 generate tolerogenic human DCs with the ability to promote Tregs. **A** Human in vitro model for the generation of human monocyte-derived DCs in the presence of Ca10 (Ca10/hmoDCs). **B** Mean fluorescence intensity (MFI) values for the surface markers HLA-DR, CD83, CD14, PD-L1 and ICOSL in conventional hmoDCs and Ca10/hmoDCs (*n* = 6–7). **C** Heatmap representation of gene expression on freshly generated conventional hmoDCs and Ca10/hmoDCs (*n* = 4). **D** Cytokine production after stimulation of hmoDCs or Ca10/hmoDCs with LPS for 18 h in cell-free supernatants (*n* = 6–7). **E** TNF-α/IL-10, IL-6/IL-10 and IL-1β/IL-1RA ratios produced by hmoDCs or Ca10/hmoDCs after 18 h of stimulation with LPS (*n* = 6–7). **F** Kinetic study of the mitochondrial oxygen consumption rate (OCR) in conventional hmoDCs and Ca10/hmoDCs by sequential addition of oligomycin (olig), carbonyl cyanide-4 (trifluoromethoxy) phenylhydrazone (FCCP) and rotenone/antimycin A (Rot/AA). Quantification of basal respiration, maximal respiration, ATP production-coupled respiration and spare respiratory capacity of DCs are included (*n* = 6 of two independent experiments). **G** Cytokines produced by allogeneic naïve CD4^+^ T cells primed by hmoDCs or Ca10/hmoDCs after 5 days quantified in cell-free supernatants (n = 8). **H** IL-10/IFN-γ and IL-10/IL-5 ratios produced by T cells primed by hmoDCs or Ca10/hmoDCs (*n* = 5–7). **I** Percentage of Tregs gated on CD4^+^ T cells by conventional hmoDCs or Ca10/hmoDCs after 5 days (*n* = 6). Representative dot plots are shown. Values are mean ± SEM. Statistical significance was determined using the Wilcoxon test (**B**–**H**) and paired Student’s *t* test (**I**). *P < 0.05, and **P < 0.01

To analyze whether Ca10 could reprogram the differentiation of monocytes into DCs, purified human monocytes from healthy donors were differentiated into DCs under conventional protocols in the presence or absence of Ca10 (Fig. 5A). Ca10/hmoDCs expressed significantly higher levels of HLA-DR, CD83, CD14 and the tolerogenic markers PD-L1 and ICOSL than conventional hmoDCs generated in the absence of Ca10 (Fig. 5B). Ca10/hmoDCs showed increased mRNA expression levels of the tolerogenic genes *PDL1*, *IDO*, *SOCS1* and *SOCS3* compared with conventional hmoDCs, suggesting a tolerogenic phenotype (Fig. 5C). After LPS stimulation, Ca10/hmoDCs produced significantly lower concentrations of the proinflammatory cytokines TNF-α, IL-6 and IL-1β as well as the anti-inflammatory cytokine IL-10 (Fig. 5D). Despite the lower production of IL-10 by Ca10/hmoDCs, the TNF-α/IL-10, IL-6/IL-10 and IL-1β/IL-10 ratios were significantly lower in Ca10/hmoDCs than in hmoDCs (Fig. 5E). These results suggested that during the differentiation process, Ca10 might endow hmoDCs with an anti-inflammatory phenotype. Functional metabolic experiments using a Seahorse bioanalyzer showed a significant decrease in basal and maximal respiration, ATP production-coupled respiration and spare respiratory capacity in freshly differentiated Ca10/hmoDCs compared with hmoDCs (Fig. 5F), suggesting lower mitochondrial OXPHOS activity in Ca10/hmoDCs. Remarkably, Ca10/hmoDCs generated T cells produced significantly lower concentrations of IFN-γ than T cells primed by conventional hmoDCs, without differences in IL-5 or IL-10 production (Fig. 5G). The IL-10/IFN-γ ratio was significantly higher when T cells were generated by Ca10/hmoDCs than when T cells were generated by conventional hmoDCs, and no differences were observed in the IL-10/IL-5 ratio (Fig. 5H). Supporting these data, Ca10/hmoDCs induced a significantly higher percentage of CD4^+^CD127^-^CD25^high^FOXP3^+^ Tregs than conventional hmoDCs (Fig. 5I). Collectively, these results indicated that Ca10 is also able to reprogram the differentiation of monocytes into DCs with anti-inflammatory and tolerogenic features.

### Ca10 does not show direct effects on human Treg generation and expansion

**Fig. 6:**
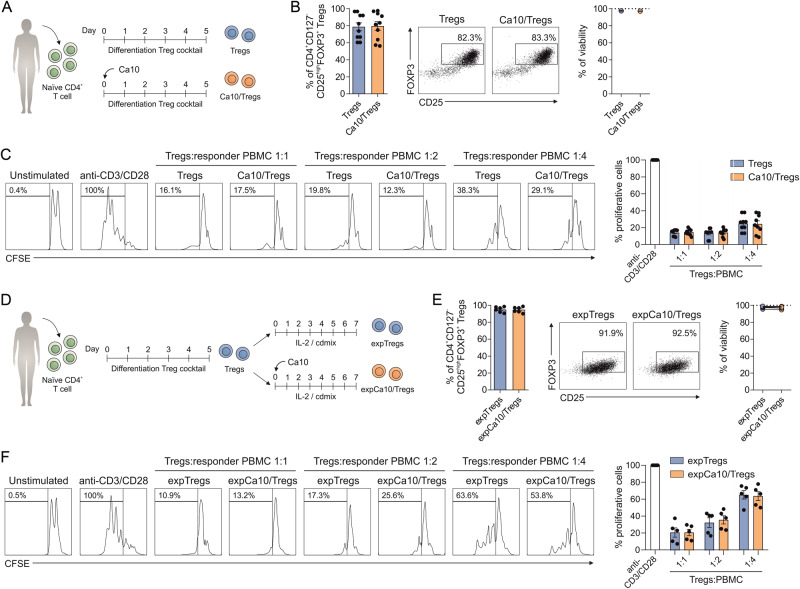
Direct effects of Ca10 on Treg generation and expansion. **A** Human in vitro model for Treg generation from naïve CD4^+^ T cells in the absence (Tregs) or presence of Ca10 (Ca10/Tregs). **B** Percentage of Tregs generated from naïve CD4^+^ T cells after 5 days. Representative dot plots are shown. Percentage of viability for the indicated conditions as determined by flow cytometry (*n* = 10). **B** Suppression assays of generated Tregs in the presence or absence of Ca10. Percentage of proliferating CD4^+^ cells after 5 days of stimulation in the presence of autologous Tregs generated from naïve CD4^+^ T cells in the presence or absence of Ca10 at different ratios (*n* = 9). **D** Human in vitro model for re-expansion of Tregs in the absence (expTregs) or presence of Ca10 (expCa10/Tregs). **E** Percentage of generated Tregs after 7 days of re-expansion. Representative dot plots are shown. Percentage of viability for the indicated conditions as determined by flow cytometry (*n* = 6). **F** Suppression assays of generated Tregs in the presence or absence of Ca10. Percentage of proliferating CD4^+^ cells after 5 days of stimulation in the presence of autologous Tregs re-expanded in the presence or absence of Ca10 at different ratios (*n* = 5). Values are mean ± SEM

To assess the potential capacity of Ca10 to regulate Treg generation by directly acting on T cells, purified naïve CD4^+^ T cells from healthy donors were differentiated under conventional protocols for Treg generation in the presence or absence of Ca10 (Fig. 6A). The percentage of generated CD4^+^CD127^−^CD25^high^FOXP3^+^ Tregs in the presence of Ca10 (Ca10/Tregs) did not show significant differences from that obtained without Ca10 following a conventional differentiation protocol for Treg generation (Fig. 6B). We did not observe differences in cell viability when comparing both assayed conditions (Fig. 6B). Importantly, these generated Ca10/Tregs and Tregs displayed similar functional suppression capacities (Fig. 6C). Next, to assess the potential direct effects of Ca10 on the expansion of already differentiated Tregs, Ca10 was added during the re-expansion of previously generated Tregs (Fig. 6D). After 7 days of re-expansion, the percentage of CD4^+^CD127^-^CD25^high^FOXP3^+^ Tregs was similar without differences in cell viability (Fig. 6E). We did not observe significant differences in the functional suppression capacity between the expanded Tregs with or without Ca10 (Fig. 6F). Collectively, these results suggest that Ca10 does not exert direct effects on the differentiation, activation and survival of Tregs under the assayed conditions.

### Ca10 is a heparan sulfate-related glycosaminoglycan driving the induction of tolerogenic DC and Treg generation

**Fig. 7:**
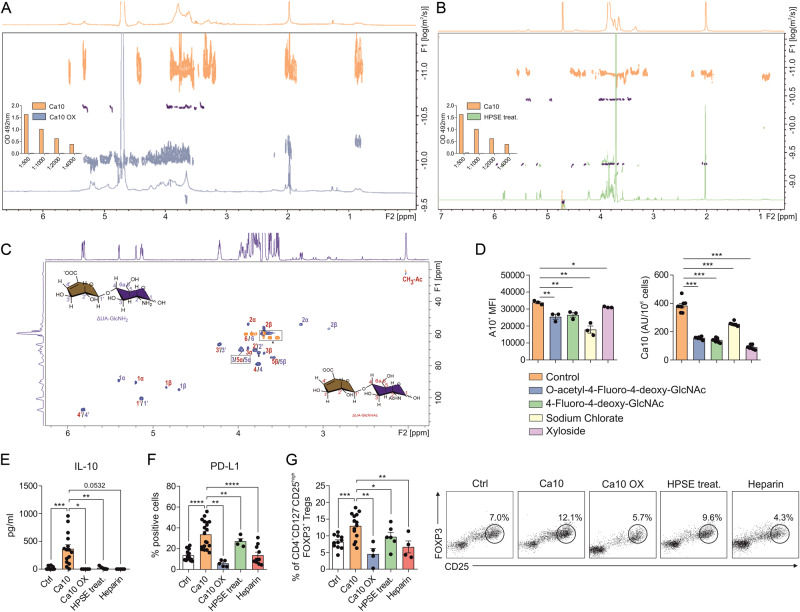
The carbohydrate structure of Ca10 is a heparan sulfate-related glycosaminoglycan driving tolerogenic DC and Treg induction. **A** Two-dimensional (2D) diffusion-ordered spectroscopy (DOSY) and one-dimensional, 1D-1H spectra of Ca10 (control, orange) and Ca10 subjected to oxidation with NaIO_4_ (Ca10 OX, blue). The 2D-DOSY spectrum of pullulan 100 kDa (dark blue) is included as a size standard; all of them were acquired at 298 K; the vertical axis corresponds to the logarithm of the diffusion coefficient. Inset, A10 mAb activity before (Ca10) and after oxidation (Ca10 OX) determined by ELISA. **B** 2D-DOSY and 1D-1H spectra of Ca10 (control, orange) and Ca10 subjected to degradation treatment with heparinase (HPSE treat., green). 2D-DOSY spectra of pullulan 100 kDa (blue) and ΔUA-GlcNAc, 0,38 kDa (dark blue) are included as size standards; all of them acquired a 303 K. Inset, A10 levels before (Ca10) and after treatment with HPSE (HPSE treat.) determined by ELISA. **C** Heteronuclear single quantum coherence spectra (HSQC) spectra of heparinase-digested Ca10 disaccharides with corresponding peak assignments (unlabeled signals correspond to buffer components tris and glycerol). **D** Mean fluorescence intensity (MFI) for A10^+^ ET cells and Ca10 concentration in supernatants after 24 h (AU/10^6^ ET cells) in the presence of glycosaminoglycan biosynthesis inhibitors O-acetyl-4-fluoro-4-deoxy-GlcNAc, 4-fluoro-4-deoxy-GlcNAc, sodium chlorate and xyloside (n = 3–7). **E** IL-10 production and **F** percentage of PD-L1^+^ human DCs unstimulated or stimulated with Ca10, Ca10 OX, or Ca10 HPSE treatment in the presence of heparin or not for 18 h (*n* = 4–10). **G** Tregs induced after 5 days of priming by control-, Ca10−, Ca10 OX- or Ca10 HPSE treatment.-stimulated human DCs or Ca10-treated human DCs in the presence of heparin (*n* = 4–6). Values are mean ± SEM. Statistical significance was determined using unpaired Student’s *t* test (**D**) and paired Student’s *t* test (**E**–**G**). *P < 0.05, **P < 0.01, and ***P < 0.001

Ca10 is a heavily glycosylated high molecular weight structure, and the carbohydrate epitope recognized by mAb A10 is sensitive to NaIO_4_ oxidation (Fig. 7A, inset), which specifically cleaves bonds between vicinal carbons bearing unsubstituted hydroxyl or amino groups [26]. Treatment of Ca10 with NaIO_4_ completely abolished the mAb A10 reactivity and resulted in significant degradation and thus molecular size reduction of the carbohydrate component, as observed by NMR diffusion ordered spectroscopy (DOSY) [27] (Fig. 7A). To gain insight into the carbohydrate structure contained in Ca10 and considering that the NMR profiles of Ca10 saccharides are consistent with glycosaminoglycans (GAGs), various enzymes cleaving glycans at different positions were used (Supplementary Table 1). Exoglycosidases had no effect on Ca10, nor did they affect PNGase-F or O-glycanase. Among all the tested enzymes, Ca10 was only degraded by the GAG-lyases of the heparinase type, indicating that the glycan structure of Ca10 is a heparan sulfate-related GAG (Supplementary Table 1). Treatment of Ca10 with heparinase II/III (HPSE treat.) completely abolished mAb A10 reactivity (Fig. 7B, inset) and hydrolyzed the carbohydrate component into low molecular weight fragments, as observed by diffusion NMR (Fig. 7B). Likewise, the NMR profile of the major fraction of the resulting disaccharide fragments upon digestion matches the profiles of ∆UA-GlcNH2 and ∆UA-GlcHNHAc, which are related to heparan sulfate-forming disaccharides (Fig. 7C and Supplementary Fig. 3a, b), confirming that the main carbohydrate moiety of Ca10 is a GAG of the heparan type. Supporting these data, ET cell growth in the presence of specific inhibitors of heparan sulfate biosynthesis showed significantly lower Ca10 surface expression and lower levels of soluble Ca10 than untreated cells (Fig. 7D). Oxidation of Ca10 with NaIO_4_ (Ca10 OX), heparinase treatment (HPSE treat.) or the presence of heparin abolished Ca10-mediated IL-10 production and significantly impaired PD-L1 expression in human DCs (Fig. 7E, F). Similarly, the generation of CD4^+^CD127^-^CD25^high^FOXP3^+^ Tregs by Ca10-stimulated DCs was significantly reduced when the DCs were previously subjected to oxidation with NaIO_4_, treated with heparinase or in the presence of heparin (Fig. 7G). Remarkably, human DCs stimulated with Ca10 and previously treated with heated pronase solution to degrade protein components in the sample (Ca10+pronase) generated a similar percentage of Tregs as Ca10-stimulated DCs (Supplementary Fig. 4a). The treatment of Ca10 with pronase only slightly reduced mAb A10 reactivity, and no significant differences were observed (Supplementary Fig. 4b). In addition, the DOSY NMR analysis demonstrated a minimal change in size (apparent logD, from −11.52 to −11.05 m^2^/s) before and after pronase digestion due to degrading the protein components but not the carbohydrate proteoglycan components (Supplementary Fig. 4c). Collectively, these results demonstrate that the generation of Tregs is mediated by the presence of the epitope recognized by the mAb A10 located in the saccharide component of Ca10.

### Patients with different adenocarcinomas show higher serum levels of a human Ca10 homolog (Ca10H) than healthy controls

**Fig. 8:**
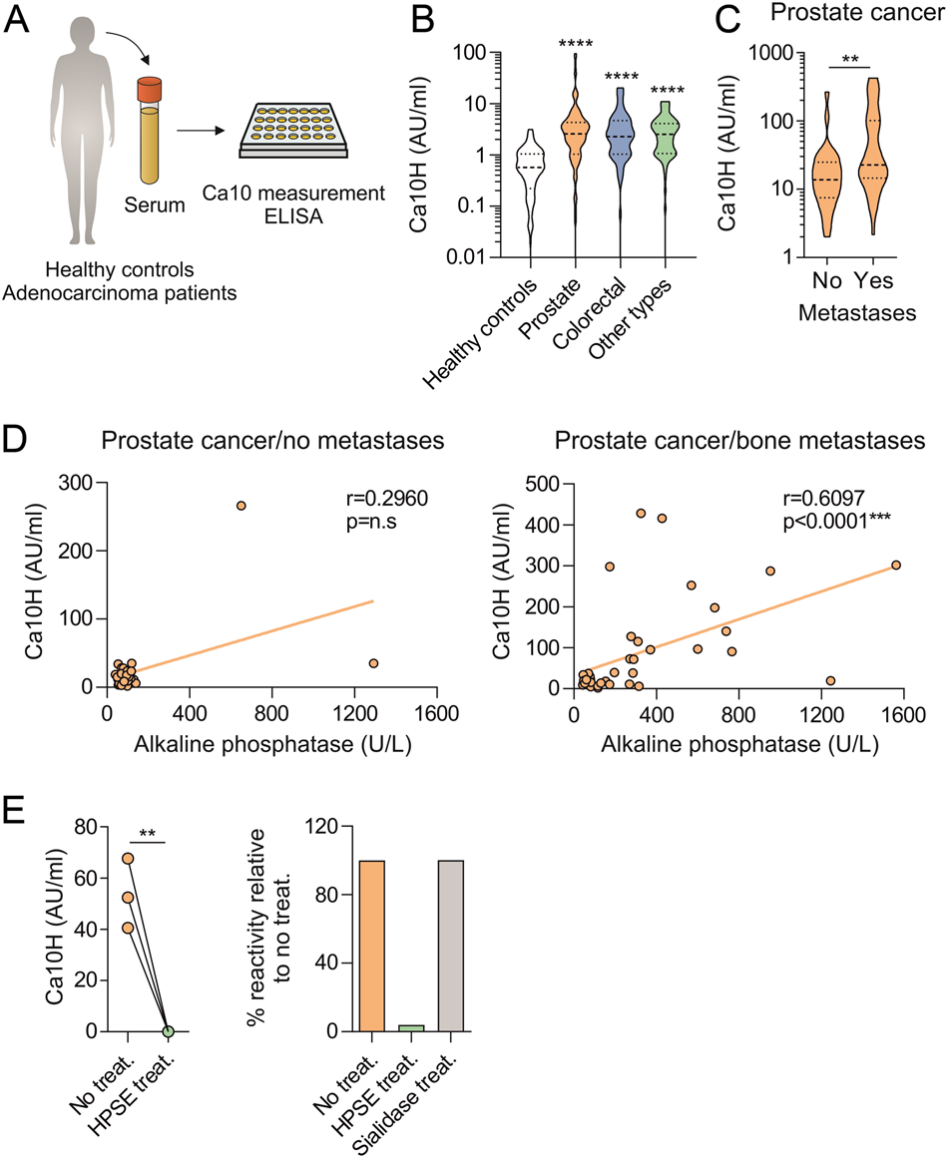
Levels of human Ca10 homolog (Ca10H) in serum from cancer patients compared with healthy controls. **A** Scheme of the protocol to determine human Ca10 homolog (Ca10H) concentrations in the serum of healthy donors (healthy controls) and cancer patients. **B** Concentrations of Ca10H in serum from healthy controls (*n* = 131) and patients diagnosed with prostate cancer (*n* = 248), colorectal cancer (*n* = 66) or other types of cancer (*n* = 71). **C** Concentrations of Ca10H in serum from prostate cancer patients with (*n* = 42) or without (*n* = 30) metastases. **D** Correlations between Ca10H and alkaline phosphatase serum levels in prostate cancer patients with or without metastases. **E** Concentrations of Ca10H in untreated, HPSE- or sialidase-treated serum from different prostate cancer patients (*n* = 3). Values are mean ± SEM. Statistical significance was determined using the Mann‒Whitney U test (**B**, **C**), Spearman’s correlation (**D**) and unpaired Student’s *t* test (**E**). **P < 0.01, and ***P < 0.001

To assess the levels of serum human Ca10 homolog (Ca10H) in cancer patients and healthy controls, the same ELISA as that used for measuring murine Ca10 levels was used as described in the Materials and Methods (Fig. 8A). Sera from patients with different types of cancer under follow-up at any stage of the disease were tested. As shown, the Ca10H levels in patients with prostate (*n* = 248), colorectal (*n* = 66) or other cancer types (*n* = 71) were significantly increased compared to those measured in healthy donors (*n* = 131) (Fig. 8B). To gain preliminary insight into the potential clinical relevance of Ca10H levels in cancer patients, different serum samples from prostate cancer patients with (*n* = 42) or without (*n* = 30) bone metastases were tested. As shown in Fig. 8C, prostate cancer patients with bone metastases showed higher levels of Ca10H than those without metastases. Supporting these data, serum Ca10H levels correlated with serum alkaline phosphatase levels in patients with bone metastases, a well-recognized bone turnover biomarker (Fig. 8D). Notably, treatment of different sera from prostate cancer patients with heparinase, but not with sialidase used as a control, completely abrogated Ca10H levels in those sera (Fig. 8E), verifying the relationship of human Ca10H to murine Ca10 and to heparan sulfate, at least in the serum of prostate cancer patients.

## Discussion

In this study, we demonstrate that the murine carbohydrate Ca10 derived from ET cells is mainly composed of heparan sulfate-related structures that promote the generation of tolerogenic human DCs, which are able to produce highly suppressive FOXP3^+^ Tregs by mechanisms partially dependent on PD-L1, IL-10, IDO, the mTOR pathway and glycolysis. Our in vivo data demonstrate positive correlations between serum levels of Ca10, tumor size and the generation of splenic Tregs in mice bearing solid ET. The administration of Ca10 to tumor-free mice also increases the proportion of splenic Tregs, which is fully impaired by the mAb A10 through the recognition of the saccharide portion of Ca10. Remarkably, we also provide evidence supporting the presence of a circulating human counterpart and show, for the first time, that serum levels of Ca10H are increased in patients suffering from different cancer types compared to those in healthy individuals. Of note, in prostate cancer patients, these levels are higher in those with bone metastases. Collectively, we reveal novel molecular mechanisms by which heparan sulfate-related structures associated with tumor cells promote the generation of functional Tregs in cancer. The discovery of this novel structural-functional relationship might well pave the way for the identification of potential novel tumor biomarkers and for the improvement or development of alternative therapeutic strategies in cancer.

Several studies convincingly demonstrated that highly suppressive Tregs are enriched in different tumors, playing a crucial role in tumor development and progression by impairing proper host antitumor immune responses [1, 8, 28–30]. Recent findings have shown that Tregs also represent a major barrier to anticancer immunity by dampening the efficacy of immune checkpoint inhibitors [31–33] or CAR-T-cell therapies [34]. Despite considerable advances in the understanding of how tumor-resident Tregs execute their suppressive functions and promote cancer progression, the mechanisms by which functional Tregs are generated and/or expanded in cancer remain incompletely understood. Malignant transformation is frequently accompanied by aberrant changes in tumor-associated surface glycoconjugates, which have a strong influence on tumor progression [20, 35]. By using ET-bearing mice, as a paradigmatic tumor model with efficient tumor escape mechanisms capable of growing in hosts with very different genetic backgrounds, we observed positive correlations between the serum levels of Ca10, tumor size and splenic Treg frequency. Such a link may suggest that this tumor-associated carbohydrate could play a role in the generation of Tregs, thus promoting tumor progression. Moreover, mice with large tumors at earlier time points displayed higher Treg numbers than those with large tumors generated at later time points. Remarkably, the i.v./i.p. administration of Ca10 significantly increased the number of splenic Tregs in ET-free mice, which was prevented by administering mAb A10. Collectively, these data confirm the effect of this tumor-associated carbohydrate on the generation of Treg cells in vivo.

To determine the potential molecular mechanisms by which Ca10 could promote Treg generation, we first studied the capacity of this tumor-associated carbohydrate to immunomodulate the phenotypic and functional features of human DCs. These cells are key players in the generation and expansion of functional Tregs in both lymphoid tissues and the TME [10]. Our data showed that Ca10 promotes human tolerogenic DCs with the capacity to generate highly suppressive functional Tregs. Mechanistically, Ca10 induced the activation of MAPK- and NF-κB-mediated signaling pathways that regulate the imprinted cytokine signature in human DCs. DCs are equipped with different Syk-coupled CLRs to sense sugar structures. Upon activation, CLRs induce several downstream signaling pathways, including the MAPK and NF-κB pathway, which under certain circumstances might promote the generation of tolerogenic DCs [12, 25, 36, 37]. In this regard, our findings suggest that Ca10 might trigger Syk-coupled CLRs and/or other receptors expressed on human DCs in a Ca^2+^-dependent manner, which in turn would initiate the abovementioned downstream signaling pathways imprinting tolerogenic features. We showed that Ca10-stimulated human DCs generate FOXP3^+^ Tregs via PD-L1, IL-10, IDO, the mTOR pathway and glycolysis, which are key factors associated with the capacity of DCs to generate Tregs and to impair antitumor immune responses [38]. Blocking PD-L1 on Ca10-stimulated DCs significantly increased the levels of IFN-γ in T-cell coculture experiments, indicating that Ca10-induced PD-L1 on human DCs is essential for the generation of suppressive Tregs vs. IFN-γ-producing T cells. Supporting all these findings, the mAb A10 impairs the capacity of Ca10-activated human DCs to generate Tregs. In addition, our data suggest that Ca10 does not exert effects on the differentiation, activation or survival of Tregs by directly acting on T cells under the assayed conditions. Metabolic reprogramming plays a key role in the control of the functional features of DCs by regulating tolerogenicity vs. immunogenicity [39, 40]. Remarkably, Ca10 enhanced glycolysis without altering mitochondrial oxidative phosphorylation, indicating that Ca10 induces metabolic reprogramming in human DCs characterized by a shift to glycolysis with significant lactate production, which might also contribute to the reported tolerogenic features. It has been previously shown that lactate impairs antitumor immunity within the TME by different mechanisms, including direct suppression of DCs [41, 42]. Tolerogenic DCs produce high levels of lactate, which suppresses the proliferation of effector T cells and induces the generation of Tregs [43, 44]. Lactate accumulation within the TME also directly supports tumor-infiltrating functional Tregs via metabolic adaptations, thus enhancing tumor survival [3]. mTOR plays a central role in the regulation of metabolic pathways in DCs, controlling cellular growth, proliferation, cytokine production and antigen presentation [45]. Thus, our data uncover that the mTOR signaling pathway and glycolysis are essential for the generation of functional Tregs by Ca10-activated human DCs.

During tumor progression, monocytes are recruited from the circulation to tumor sites, and the signals provided by the TME influence their capacity to differentiate into tolerogenic DCs, which promote Treg generation while decreasing effector T cells within the tumor [46, 47]. Our data showed that Ca10 is able to reprogram the differentiation of monocytes into DCs displaying a tolerogenic phenotype with increased levels of *PDL1*, *ICOSL*, *IDO*, *SOCS1* and *SOCS3* and reduced capacity to produce cytokines upon LPS stimulation. Ca10/hmoDCs also induce a higher proportion of Tregs and lower proportion of IFN-γ-producing T cells than conventional DCs, which might well represent a mechanism of tumor escape that also contributes to tumor progression [1, 4]. As observed for Tregs, Ca10 does not appear to have a direct effect on tumor cells. The in vitro proliferation of suspension ET cells was not affected by the presence of Ca10 (data not shown). Ca10 did not directly affect the migration or proliferation capacity of other adherent tumor cells (Supplementary Fig. 5a–c).

Previous studies have shown that Ca10 is a highly glycosylated ET cell surface macromolecule defined by monoclonal antibody A10 binding [22, 23]. By using NMR and chemoenzymatic structural analysis, we show here that the carbohydrate structure of Ca10 is mainly a heparan sulfate-related glycosaminoglycan. Interestingly, different pharmacological inhibition experiments and functional assays demonstrated that this GAG in Ca10 is essential for both mAb A10 binding and for the induction of tolerogenic DCs with the capacity to generate functional Tregs. Although different studies previously showed that DCs within the TME may become tolerogenic and favor tumor progression through different mechanisms, in this study, we demonstrate, for the first time, that a tumor-associated heparan sulfate-related GAG promotes human tolerogenic DCs that are able to generate highly suppressive Tregs. Importantly, we were also able to demonstrate the presence of a circulating Ca10 homolog in human serum with the same assay used for murine Ca10. Of note, the serum levels of Ca10H were significantly higher in patients suffering from prostate, colorectal and other cancer types compared to healthy donors, suggesting that Ca10H plays a role in cancer. Although its relevance in human tumor development and progression is unknown, we found an initial correlation between serum Ca10H levels and alkaline phosphatase levels in prostate cancer patients with bone metastases. Interestingly, the recruitment and expansion of Tregs in the bone marrow of prostate cancer patients with bone metastases has been described [48]. Whether Ca10H can act by promoting the induction of tolerogenic DCs and Tregs, as we show here for its murine counterpart, is still unknown, but results in prostate cancer patients could point in that direction.

In summary, this study demonstrates the involvement of a tumor-associated heparan sulfate structure in the induction of tolerogenic responses and thus plays a potential role in tumor escape and progression.

### Supplementary information

Supplementary Figure 1

Supplementary Figure 2

Supplementary Figure 3

Supplementary Figure 4

Supplementary Figure 5

Unprocessed original Western blots

Supplementary figure and table legend
