# Supplementary figures and images for: A tumor-associated heparan sulfate-related glycosaminoglycan promotes the generation of functional regulatory T cells
Source: Cell Mol Immunol. 2023 Nov 22;20(12):1499–512. doi: 10.1038/s41423-023-01096-9 (PMC10687014; doi:10.1038/s41423-023-01096-9)

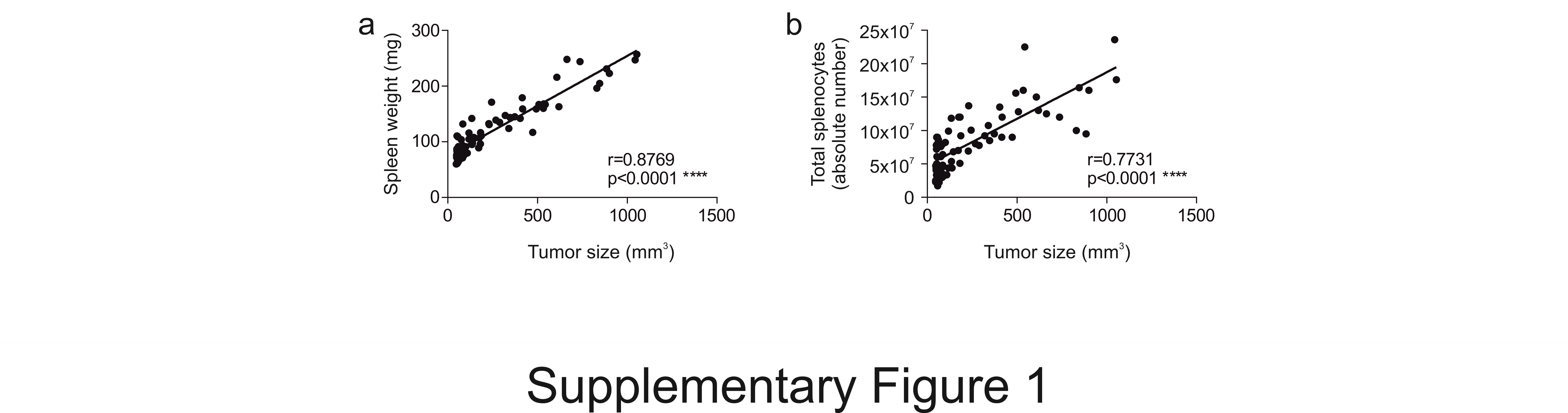

Supplement: Supplementary file 1 — Supplementary Figure 1 [file 41423_2023_1096_MOESM1_ESM.tif]

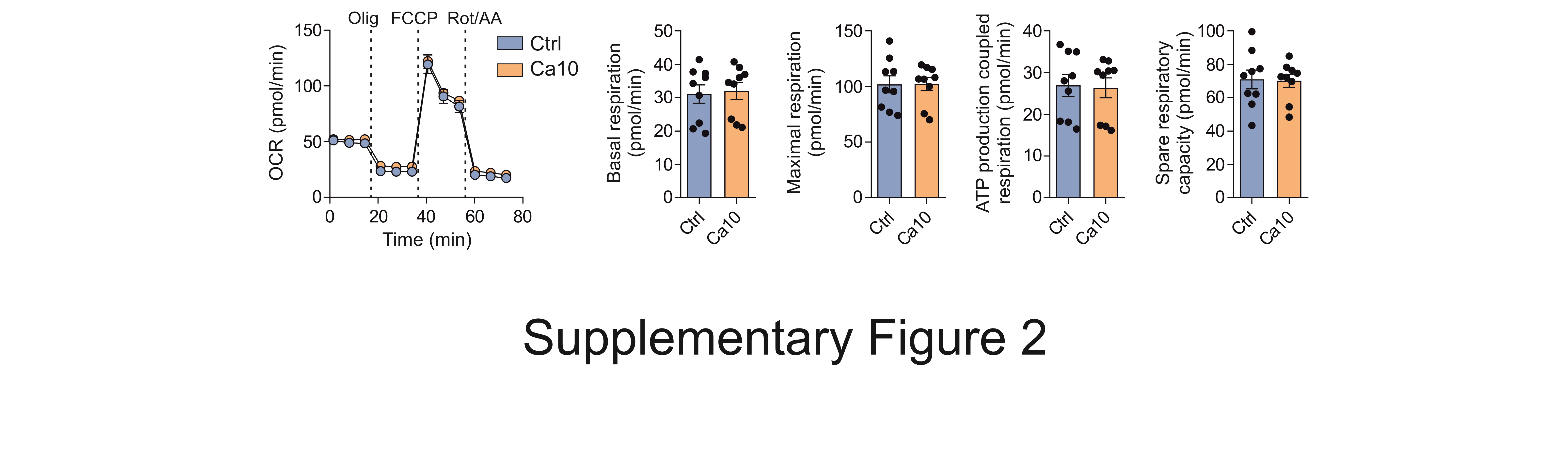

Supplement: Supplementary file 2 — Supplementary Figure 2 [file 41423_2023_1096_MOESM2_ESM.tif]

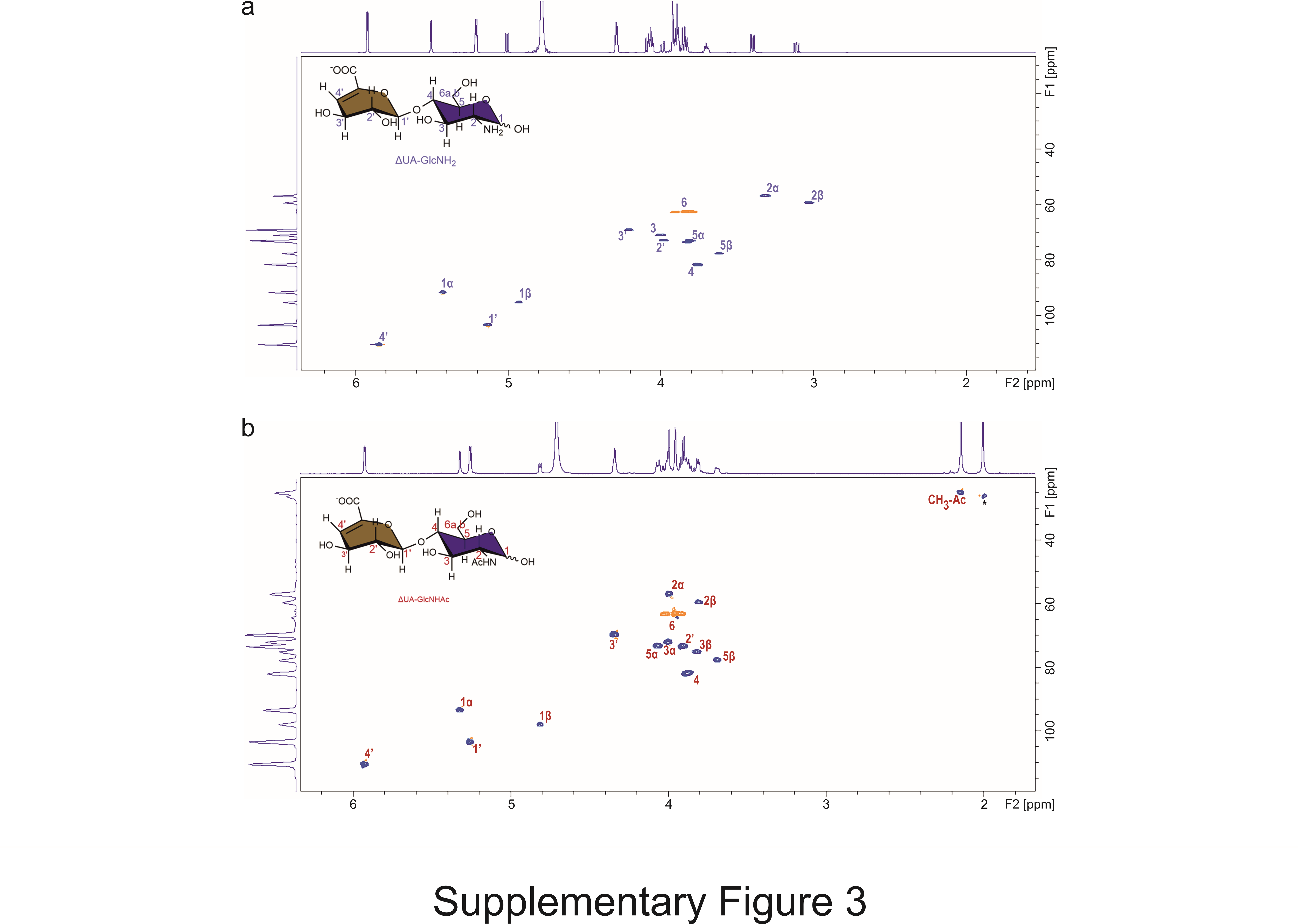

Supplement: Supplementary file 3 — Supplementary Figure 3 [file 41423_2023_1096_MOESM3_ESM.tif]

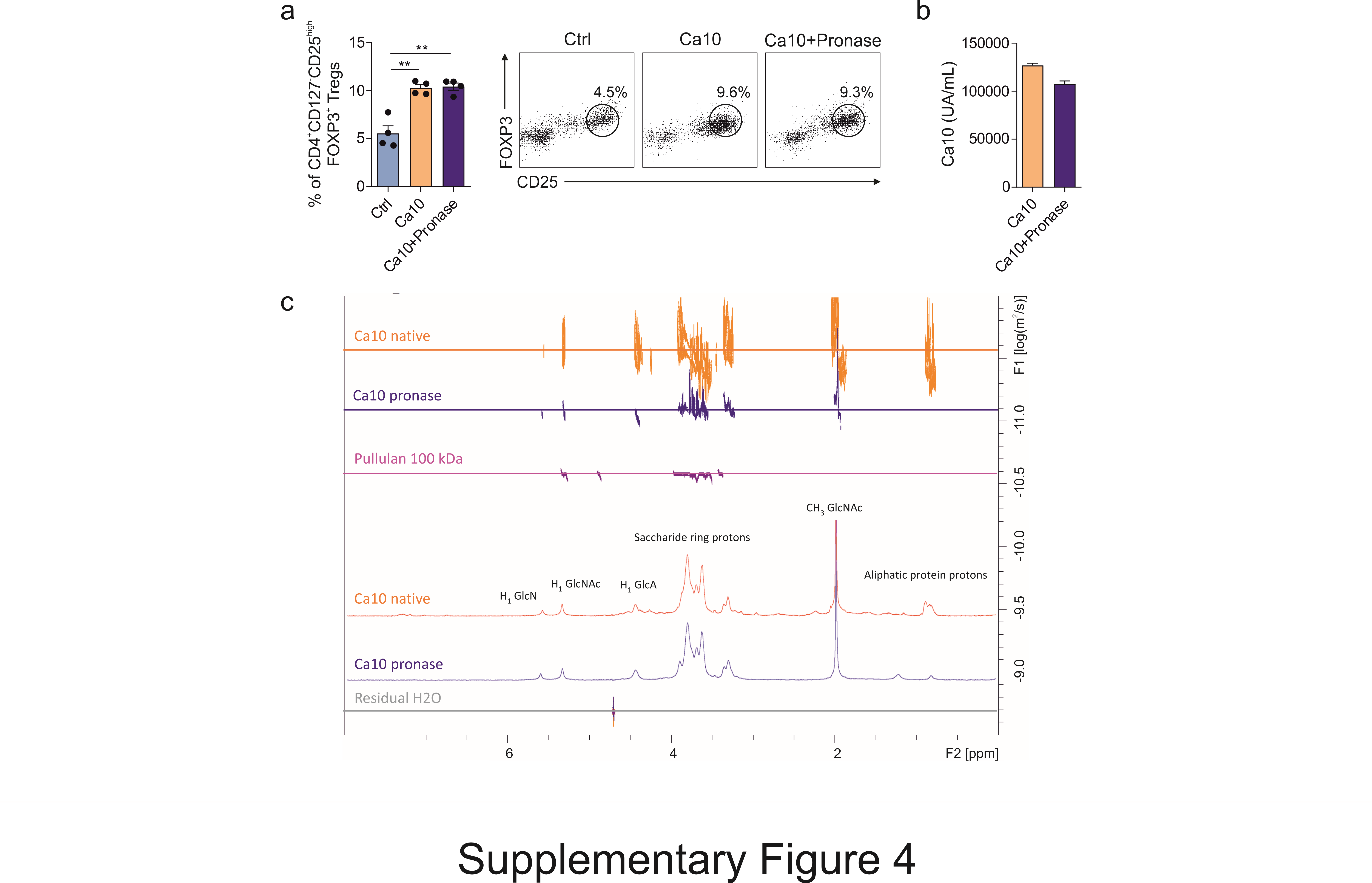

Supplement: Supplementary file 4 — Supplementary Figure 4 [file 41423_2023_1096_MOESM4_ESM.tif]

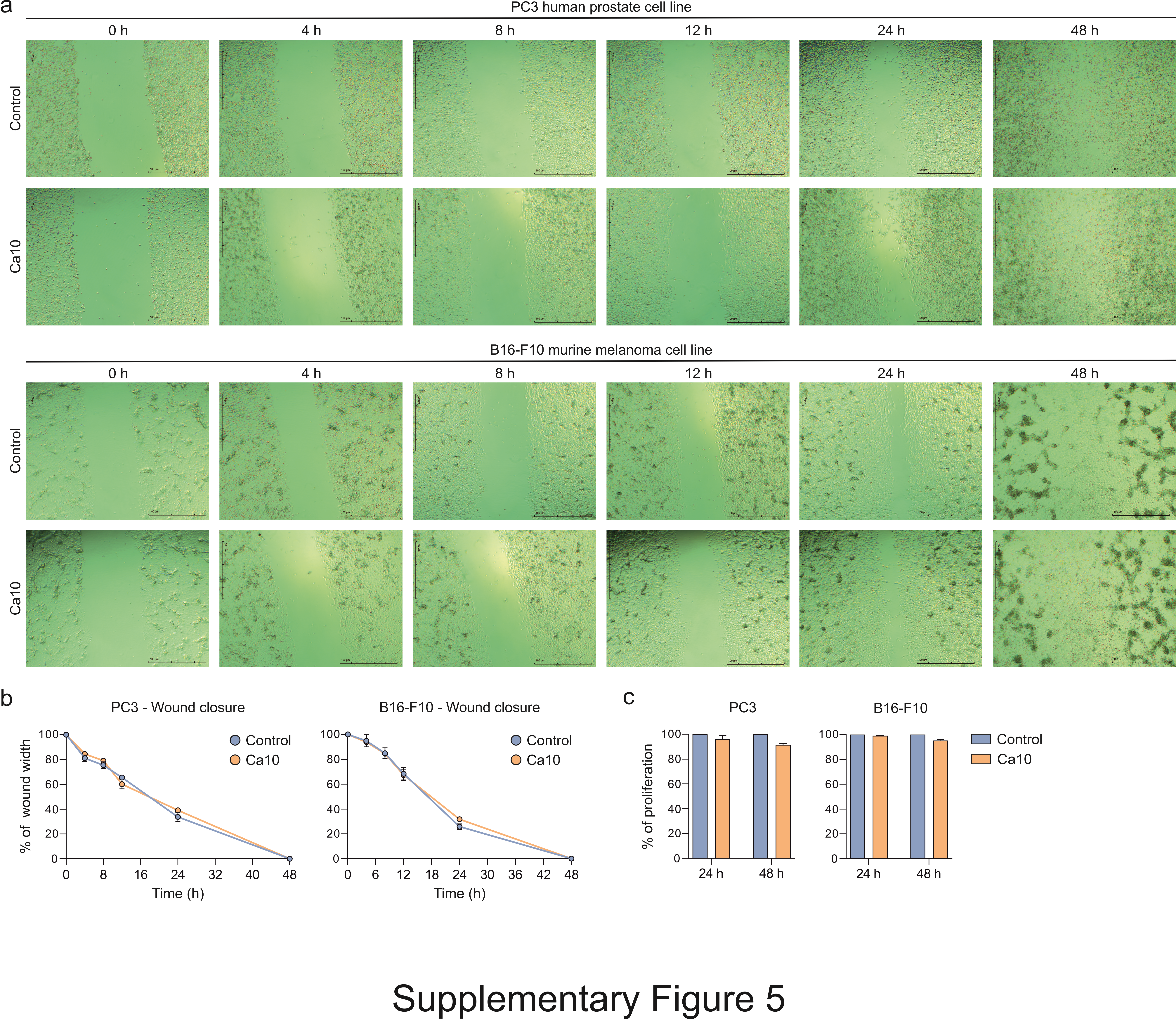

Supplement: Supplementary file 5 — Supplementary Figure 5 [file 41423_2023_1096_MOESM5_ESM.tif]

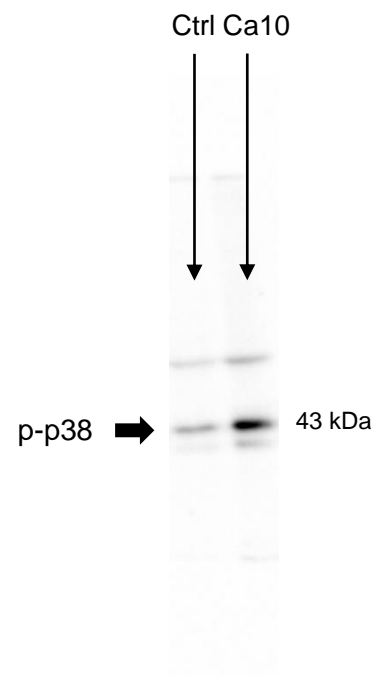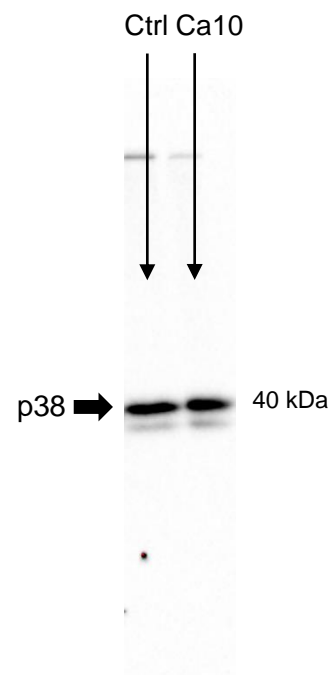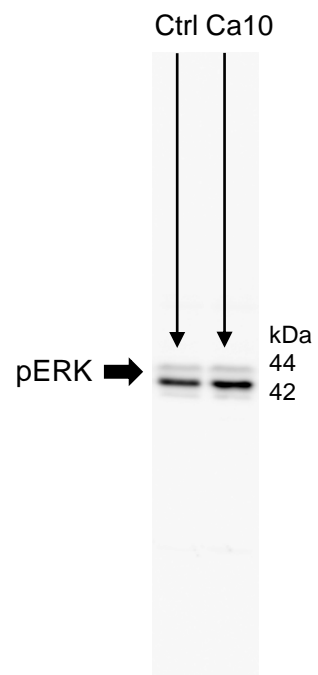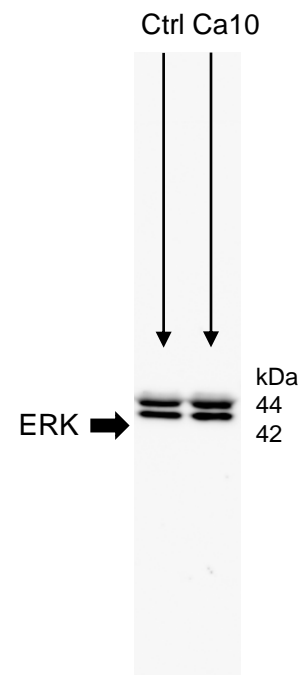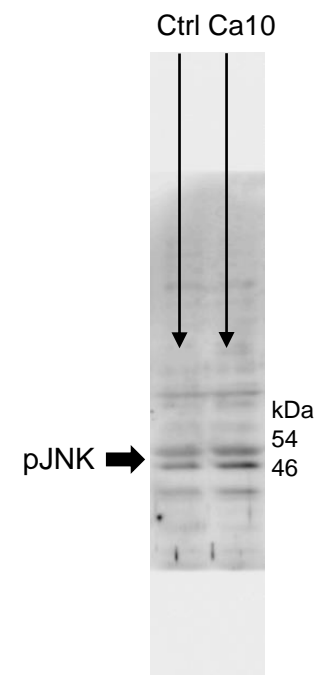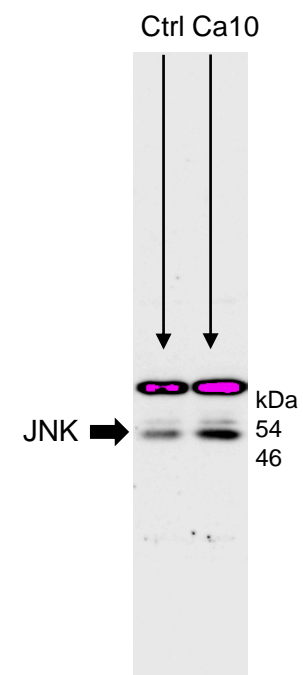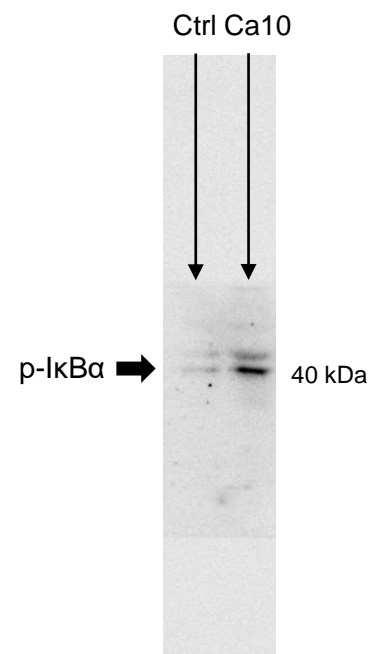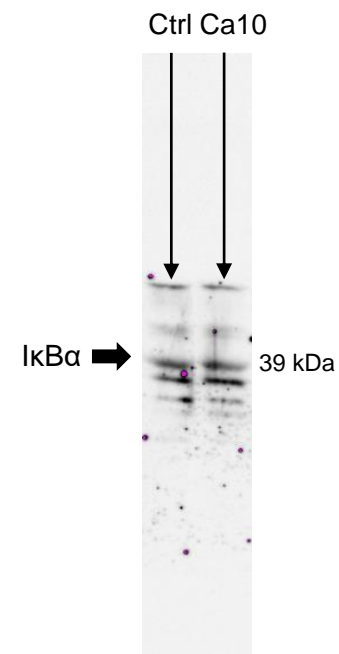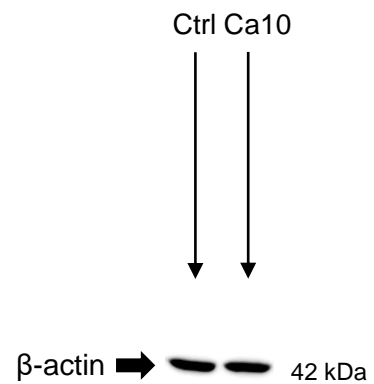

Supplement: Supplementary file 6 — Unprocessed original Western blots [file 41423_2023_1096_MOESM6_ESM.pdf]
