# Supplementary material for: A tumor-associated heparan sulfate-related glycosaminoglycan promotes the generation of functional regulatory T cells
Source: Cell Mol Immunol. 2023 Nov 22;20(12):1499–512. doi: 10.1038/s41423-023-01096-9 (PMC10687014; doi:10.1038/s41423-023-01096-9)
Supplement: Supplementary file 7 — Supplementary figure and table legend [file 41423_2023_1096_MOESM7_ESM.docx]

**Supplementary Figure 1. a**, Correlation of spleen weight with tumor size from Ehrlich tumor-bearing mice. **b**, Correlation of the total number of cells in the spleen of Ehrlich tumor-bearing mice with tumor size. Values are the mean ± SEM of 79 individual mice of individual mice of two independent experiments. Statistical significance was determined using the Spearman test. *****P* < 0.0001.

**Supplementary Figure 2.** Kinetic study of the mitochondrial oxygen consumption rate (OCR) in Ctrl- or Ca10-stimulated human DCs by sequential addition of oligomycin (olig), carbonyl cyanide-4 (trifluoromethoxy) phenylhydrazone (FCCP), and rotenone/antimycin A (Rot/AA). Quantification of basal respiration, maximal respiration, ATP production-coupled respiration and spare respiratory capacity of human DCs are included (n=9 of three independent experiments). Values are mean ± SEM.

**Supplementary Figure 3.** **a-b**, HSQC spectra of heparan sulfate disaccharide standards ∆UA-GlcN (**a**) and ∆UA-GlcNAc (**b**) with the corresponding peak assignments.

**Supplementary Figure 4. a**, Percentage of Tregs gated on CD4^+^ T cells after 5 days of coculture with unstimulated, Ca10- or Ca10+Pronase-treated human DCs (n = 4). Representative dot plots are shown. **b**, A10 mAb levels before (Ca10) and after pronase treatment (Ca10+pronase) determined by ELISA (n = 4). **c**, Overlaid DOSY of Ca10 Native extract (orange) and after pronase treatment (blue). The DOSY trace of pullulan 100 kDa (purple) is included as a molecular size reference. The corresponding 1D-proton trace of the DOSY acquired with 50% gradient strength is included. Selected proton assignments are labeled. Values are mean ± SEM.

**Supplementary Figure 5. a**, PC3 human prostate and B16-F10 mouse melanoma cell lines treated with and without Ca10. Representative pictures at 0, 4, 8, 12, 24 and 48 h are shown. Scale bars (100 µm) were added for wound width measurement. **b**, Quantification of wound closure over 48 h by untreated (blue) cells and cells treated with Ca10 (orange). The percentage of wound closure relative to time 0 h (100%) is shown (n = 9 width measurements per time). **c**, PC3 human prostate and B16-F10 mouse melanoma cell line proliferation rate in untreated (blue) cells and cells treated with Ca10 (orange) for 24 and 48 h relative to that in untreated cells (control) (n = 3). Values are mean ± SEM.

**Supplementary Table 1. Enzymatic digestion of Ca10 preparations.**

Summary of the different enzymes used for the enzymatic digestions of Ca10 preparation and the specific substrates used as controls. Activity represents the effect of these digestions on control and Ca10 preparations, analyzing the sugars derived from the digestion by NMR or Ca10 sandwich ELISA evaluation. (+) represents positive digestion by the enzyme used, (-) represents that no enzymatic digestion occurred.

|  | Activity | |
| --- | --- | --- |
| Enzyme | Control | Ca10 |
| PNGase-F* | ++++ | - |
| O-Glycanase* | ++ | - |
| *β*-Acetylglucosaminidase 73A | ++++ | - |
| Glucosidases (*α*-, *β*-) | ++++ | - |
| Galactosidases (*α*-, *β*-) | ++++ | - |
| *α*-Manosidase | +++ | - |
| Sialidase | ++++ | - |
| *α*-Fucosidase | ++++ | - |
| Chitosanase 8B | ++++ | - |
| Heparinase III | +++ | ++ |
| Heparinase II | +++ | + |
| Chondroitinase ABC | ++++ | + |
